# Microbial Community Shifts and Nitrogen Utilization in Peritidal Microbialites: The Role of Salinity and pH in Microbially Induced Carbonate Precipitation

**DOI:** 10.1007/s00248-025-02532-1

**Published:** 2025-04-22

**Authors:** Yunli Eric Hsieh, Sung-Yin Yang, Shao-Lun Liu, Shih-Wei Wang, Wei-Lung Wang, Sen-Lin Tang, Shan-Hua Yang

**Affiliations:** 1 Systems Biology and Mathematical Modeling Group, Max Planck Institute of Molecular Plant Physiology, Potsdam, Germany; 2 Bioinformatics Department, Institute of Biochemistry and Biology, University of Potsdam, Potsdam, Germany; 3 School of BioSciences, The University of Melbourne, Parkville, Australia; 4 Department of Aquatic Biosciences, National Chiayi University, Chiayi, Taiwan; 5 Department of Life Science & Center for Ecology and Environment, Tunghai University, Taichung, Taiwan; 6 Department of Geology, National Museum of Natural Science, Taichung, Taiwan; 7 Department of Biology, National Changhua University of Education, Changhua, Taiwan; 8 Biodiversity Research Center, Academia Sinica, Taipei, Taiwan; 9 Institute of Fisheries Science, National Taiwan University, Taipei, Taiwan

**Keywords:** Microbially induced carbonate precipitation, Peritidal microbialites, Microbial community, Salinity, pH

## Abstract

Microbialites have the potential to record environmental changes and act as biosignatures of past geochemical conditions. As such, they could be used as indicators to decipher ancient rock records. Modern microbialites are primarily found in environments where competitors and destructors are absent or where biogeochemical conditions favor their continuous formation. Many previous studies have essentially focused on the role of photosynthetic microbes in controlling pH and carbonate speciation and potentially overlooked alternative non-photosynthetic pathways of carbonate precipitation. Given that microbial activity induces subtle geochemical changes, microbially induced carbonate precipitation (MICP) can involve several mechanisms, from extracellular polymeric substances (EPS), sulfate reduction, anaerobic oxidation of methane, to nitrogen cycling processes, such as ammonification, ureolysis, and denitrification. Moreover, the peritidal zone where temperate microbialites are mostly found today, is under the influence of both freshwater and seawater, arguing for successive biogeochemical processes leading to mineral saturation, and questioning interpretations of fossil records. This study investigates microbialites in three tide pools from the peritidal zone of Fongchueisha, Hengchun, Taiwan, to address the influence of salinity on microbial community composition and carbonate precipitation mechanisms. Microbial samples were collected across varying salinity gradients at multiple time points and analyzed using next-generation sequencing (NGS) of bacterial 16S and eukaryotic 18S rRNA genes. Our results indicate that dominant bacterial groups, including Cyanobacteria and Alphaproteobacteria, were largely influenced by salinity variations, albeit pH exhibited stronger correlation with community composition. Combining our results on geochemistry and taxonomic diversity over time, we inferred a shift in the trophic mode under high salinity conditions, during which the use of urea and amino acids as a nitrogen source outcompetes diazotrophy, ureolysis and ammonification of amino acids reinforcing carbonate precipitation dynamics by triggering an increase in both pH and dissolved inorganic carbon.

**Supplementary Information:**

The online version contains supplementary material available at 10.1007/s00248-025-02532-1.

## Introduction

Lithified deposits induced by microbial activity (microbialites) represent the most continuous and ancient record of life on Earth, having persisted as fossils for at least 3.43 billion years [1, 2]. Modern microbialites are became more and more confined to areas where competitors and destructors are absent or where the biogeochemical conditions are favorable for their consistent growth [3]. They can be found in various aquatic environments, ranging from freshwater, marine to hypersaline condition, also including more remote and confined ecosystems such as caves and hot springs [4]. Many recent studies on modern microbialites were conducted in coastal and inland areas, whereas microbialites forming in the intertidal zone under freshwater and saline conditions represent a distinct type that has received little attention [5–7]. Such microbialite formations are characterized by their proximity to the ocean: supratidal concretions receive freshwater inflow from seeps, those in the tidal flat endure a mix of freshwater seepage while concretions in the lowermost intertidal zone are in close connection with the ocean [8]. The recent discovery of peritidal microbialites across three continents suggests that they have been overlooked and may be more common along coastlines than previously thought [3].

Microbialites are formed by the lithification of mat, either through agglutination of detrital sediments or increased chemical precipitation at the surface [9]. In both ways, there are several potential microbially induced carbonate precipitation (MICP) involved, including extracellular polymeric substance (EPS), photosynthesis [10], sulfate reduction [11], anaerobic oxidation of methane [12], and nitrogen cycle which are ammonification [13], ureolysis [14], and denitrification [15]. According to different environments, microbialites may be constituted by different portions of metabolism pathways. In freshwater systems, Cyanobacteria and other photosynthetic microbes dominate, whereas in marine systems, photosynthesis, sulfate reduction, ureolysis, and anaerobic oxidation of methane are more prominent [16]. Recently, ureolytic bacteria were reported to increase alkalinity and thereby foster rates of carbonate precipitation in highly saline environments [17].

Microbial mat and EPS, produced by a diverse range of microorganisms, including Cyanobacteria, diatoms, microalgae, bacteria, and archaea, serve as foundational substrates for sediment trapping and mineral precipitation. Additionally, other microbial metabolic pathways mechanistically inducing carbonate precipitation usually involve an increase in alkalinity and DIC production [16]. For example, photosynthesis consumes CO₂ and HCO₃⁻ and releases OH⁻ into the surrounding environment, resulting in elevated pH levels [18]. Ureolysis involves the hydrolysis of urea into NH₄⁺ and CO₂, followed by the further hydrolysis of NH₄⁺, which produces OH⁻ and subsequently raises pH, as well as increases bicarbonate and carbonate concentrations [19]. Denitrification contributes to alkalinization through the consumption of H^+^, while processes such as ammonification, sulfate reduction, and methane oxidation increase the HCO_3_^−^ levels, which facilitating the CaCO_3_ precipitation [16]. Since microbial activity induces slight changes in geochemical conditions, leading to mineral saturation, and given that modern microbialites in the peritidal zone are influenced by both freshwater and seawater, this raises a question: Has research overly focused on photosynthetic microbes while overlooking carbonate precipitation mechanisms that may arise due to the activity of other microbes? Historically, studies have primarily emphasized photosynthetic microbes, which may have led to insufficient attention being given to potential carbonate precipitation mechanisms driven by other microbial activities.

Cyanobacteria are considered as the main EPS producers in most types of microbialites environments. Interestingly, a study by Campbell et al. [20] showed that salinity influences not only the Cyanobacteria diversity composition but also their gene expression on EPS formation. In saline environments, other microorganisms are the major contributors to the carbonate minerals precipitation [16]. Here, we successively address the role of salinity in modulating EPS production by Cyanobacteria and other microorganisms, the concomitant environmental factors that exert selection on microbial community composition, and how this in turn determines prevalent pathways of MICP. For this, we collected microbialite samples from three tide pools in the Fongchueisha area of Hengchun, spanning from the freshwater-influenced supralittoral zone to the seawater-influenced lower intertidal zone, over the course of a year. We analyzed the V8–V9 region of the 18S rRNA gene for eukaryotes and the V6–V8 region of the 16S rRNA genes for bacteria using next-generation sequencing to understand the temporal and spatial variations in the composition of microbialites.

## Methods

### Microbialite Field Sampling

Modern microbialites were documented in the peritidal zone of the Fongchueisha area in Hengchun, located in southern Taiwan. This region, which is characterized by a tropical monsoon climate, a long and varied coastline, and diverse ecological habitats, including limestone terrain and coral reefs, appears to be the only location in Taiwan where such microbialite structures were identified.

**Fig. 1:**
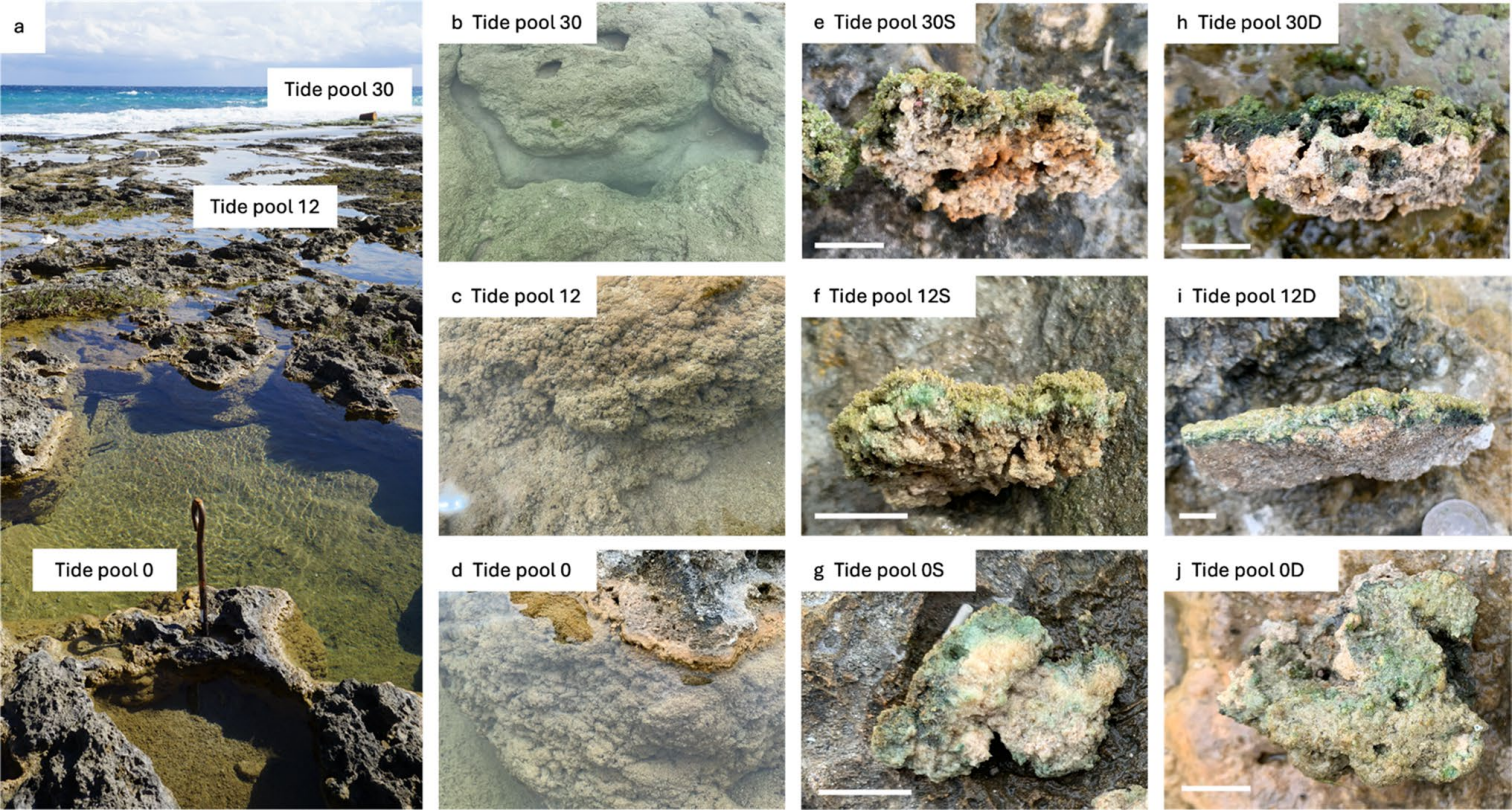
Tide pools and samples of the modern microbialites. **a** The picture of tide pools at the sampling site. **b**, **c**, **d** Each tide pool. **e**, **f**, **g** Surface microbialite sample in each tide pool. **h**, **i**, **j** Bottom microbialite sample in each tide pool. Bar indicates 1 cm

For this study, microbialites were sampled at Fongchueisha, Hengchun, Taiwan (24° 94′ 51.1′′ N, 120° 84′ 25.8′′ E; Fig. 1). Sampling was conducted along the intertidal zone from land to sea at three tide pools located in the upper (tide pool 0), middle (tide pool 12), and lower (tide pool 30) intertidal zones, with sample collections in June, August, and October 2019, and January and March 2020. For each tide pool, microbialites were sampled from both the surface (S) and bottom (D) of the pools. Following collection, some samples were preserved at 4 °C and transported to the laboratory, where they were stored at − 80 °C for DNA extraction. Additional samples were preserved in a fixative solution (2.5% glutaraldehyde, 4% paraformaldehyde, 0.1 M phosphate buffer) and stored in a dark, cool environment for subsequent electron microscopy analysis.

For environmental parameters, a YSI Pro Plus portable multiparameter water quality instrument (YSI, OH, USA) was used to measure temperature, salinity (PSU), total dissolved solids (TDS), suspended particulate concentration (SPC), dissolved oxygen (DO), and pH along the transect from the freshwater source at the upper intertidal zone (tide pool 0) to the lower intertidal zone (tide pool 30) while sampling for the microbialites. The environmental raw data is in supplementary Table S1.

### Scanning Electron Microscope

Sections from the interior of microbialite samples were carefully excised using sterilized blades, and microbial cell morphologies were analyzed using scanning electron microscopy (SEM) at the Institute of Plant and Microbial Biology, Academia Sinica. Following fixation, the samples were rinsed with a phosphate buffer three times for 10 min each. Next, the fixed samples were submerged in 1% (w/v) osmium tetroxide for 4 h and then washed three times with a phosphate buffer for 2 h each. Dehydration was performed using a graded ethanol series: 30% for 1 h, 50% for 1 h, 70% for 1 h, 85% for 2 h, 95% for 2 h, and 100% for 2 h, followed by overnight treatment in 100% ethanol. The samples were subsequently dried using a Hitachi HCP- 2 critical point dryer (Hitachi, Ltd., Tokyo, Japan) to preserve the soft organic structures, coated with gold using a Hitachi E- 1010 ion sputter coater (Hitachi, Ltd., Tokyo, Japan), and examined using an FEI Quanta 200 scanning electron microscope (FEI company, OR, USA) at 20 kV.

### Microbialite DNA Extraction, PCR, and Amplicon Sequence Analysis

Microbialite DNA from each sample was extracted using a PowerSoil DNA isolation kit (Qiagen, Germany). The bacterial community structure was investigated by amplifying the V6-V8 region of the 16S rRNA genes using primer sets 968 F (5′-AACGCGAAGAACCTTAC- 3′) and 1391R (5′-ACGGGCGGTGWGTRC- 3′) [21]. The PCR protocol entailed 30 cycles comprising an initial step of 94 $^\circ{\rm C}$ for 5 min, followed by 94 $^\circ{\rm C}$ for 30 s, 52 $^\circ{\rm C}$ for 20 s, 72 $^\circ{\rm C}$ for 45 s, and finally 72 $^\circ{\rm C}$ for 10 min. The resulting PCR products, approximately 423 bp in size, were purified using the MinElute Gel Extraction Kit (QIAGEN). The library preparation followed the Illumina suggested protocol. Subsequently, up to 90 V6-V8 amplicon libraries were prepared and subjected to quantitative PCR (qPCR) to verify the uniformity of each library prior to pooling. Sequencing of the pooled libraries was performed on an Illumina MiSeq V2 platform using the MiSeq Reagent Kit V3 (paired end [PE]; 2 × 300 bp), conducted by Biotech Ltd., Taiwan.

Amplicon sequence analysis was performed utilizing the Quantitative Insights Into Microbial Ecology 2 (QIIME 2 v 2022.2) software [22]. Primers targeting the V6-V8 regions were removed using cutadapt (version 4.0) [23]. Following primer removal, sequences were denoised using the DADA2 algorithm, which included quality filtering, trimming, and chimera removal [24]. The qualified amplicon sequence variants (ASVs) were taxonomically classified using the classifier-consensus-vsearch plugin [25], against the SILVA 138 NR99 database [26, 27].

To ascertain the community profile of eukaryotes, amplicons of the V8-V9 region of the 18S rRNA gene, approximately 315 bp in length, were generated using the primer set 1195 F (5′-AACAGGTCTGTGATG- 3′) [28] and 1510R (5′-CCTTCYGCAGGTTCACCTA- 3′) [29]. The PCR conditions were consistent with those previously described. Following sequencing on the MiSeq platform, downstream bioinformatic analyses were performed similarly to those mentioned above, with the exception that length trimming was adjusted to 206 bp for forward reads and 204 bp for reverse reads. The SILVA 138 NR99 database [26, 27] was used for taxonomic assignment. To enhance the resolution of the ASV abundance table, ASV sequences in both eukaryotes and bacteria were further clustered with the K-mer-based taxonomic (KTU) clustering algorithm [30].

### Statistical Analyses

Alpha diversity indices, Shannon, Simpson, and Fisher, were computed for all samples. Correlations between these indices and environmental factors were assessed using Spearman correlation test. Canonical correspondence analysis (CCA) (R package vegan [31]) was applied to detect relation between microbial composition (bacteria and eukaryotic) and environmental factors. Non-metric multidimensional scaling (NMDS) using Bray–Curtis similarity of KTU composition was employed to rank the bacteria and eukaryotic communities, and a permutational multivariate analysis of variance (PERMANOVA) was conducted to evaluate differences among the tide pools using vegan package in R [31].

The most dominant/abundant families identified within the phylum Cyanobacteria identified in the samples was subjected to phylogenetic analysis using MEGA 11 [32]. Sequence alignments were performed using the ClustalW algorithm [32], followed by maximum likelihood phylogenetic inference with 500 bootstrap replicates. For microbial community interaction analysis, we utilized FastSpar v1.0 [33] to estimate correlation. This tool quantifies correlations between KTUs at the family level by using the SparCC algorithm [34], determining the statistical significance of these correlations using bootstrap procedures. The resultant correlation and p-value matrices generated by FastSpar were employed to construct a network of significant correlations (*p* < 0.05) for each sample. Co-occurrence networks were created using the igraph [35] package in R software [36], consisting of undirected weighted networks based on statistically significant correlation values greater than 0.8. These networks were visualized using Cytoscape (version 3.10.0) [37]. Network analysis tools within Cytoscape calculated network properties, and networks from samples at identical depths were integrated using the “merge network” function. Nodes not directly connected to the most abundant cyanobacterial family were filtered out to refine the network structure.

Random forest, a machine learning method, was employed to construct a classification model of bacterial composition in tide pools using family-level abundance data as predictors [38]. The analysis was conducted using the RandomForest R package with 1000 trees and default parameters. Predictor importance was evaluated based on the average decrease in the Gini index, with the bacterial family showing the highest index considered the most influential in distinguishing among tide pools.

### Functional Pathway Analysis of Bacterial Community

To identify bacteria that differed in abundance between tide pools 0 and 30, the linear discriminant analysis (LDA) effect size (LEfSe) analysis [39] in genus-level was conducted using the microbiomeMarker package in R [40]. LEfSe analysis was used to identify genera with significant differences in normalized relative abundances, with linear discriminant analysis (LDA) quantifying the effect size for each genus. Genera with an LDA score exceeding 2.0 (*p* < 0.05) were considered significant. PICRUSt2 [41] was then employed to predict the potential functions of bacterial taxa showing significant abundance differences between the tide pools 0 and 30 as identified by LEfSe. KTUs associated with these genera were aligned to reference 16S rRNA sequences using HMMER [42]. Subsequently, EPA-NG [43] was utilized to determine the optimal placement of KTUs within the reference tree, and GAPPA [44] was used to generate a new phylogenetic tree. The new phylogenetic tree was then used to predict the copy numbers of enzyme classification (EC) numbers for each KTU through hidden-state prediction approaches [45]. The predicted EC number abundances for each sample were calculated and normalized by read depth. MetaCyc pathway abundances were then calculated based on the EC number abundances from each sample. Ggpicrust2 [46] was applied for a downstream analysis of pathway abundances. Differential abundance testing of pathways across different tide pools was performed using the LinDA approach [47], with *p*-values corrected for multiple hypothesis testing using the Benjamini–Hochberg procedure [48]. The top 50 pathways with significant abundance differences between tide pools 0 and 30 were presented in a heatmap.

## Results

### Morphological Variations of Microbialites in Tide Pools

In this study, three tide pools had different environmental conditions (Table S1). The tide pool 30, which was most close to the sea, in general had higher salinity, DO, TDS, and SPC than other pools, especially in the bottom. Regarding pH, although pH in the tide pool 30 was higher than other pools, the pH in the tide pool 12 had the largest range. Observations of mat thickness and pigment abundance (Fig. 1) indicated that microbialite development was more pronounced in areas proximal to land and freshwater sources, with a noticeable reduction in microbialite thickness and pigmentation toward the seaward margin. These findings suggest that the relative contribution of seawater versus freshwater may be a critical factor influencing microbialite formation in the peritidal zone of Fongchueisha.

**Fig. 2:**
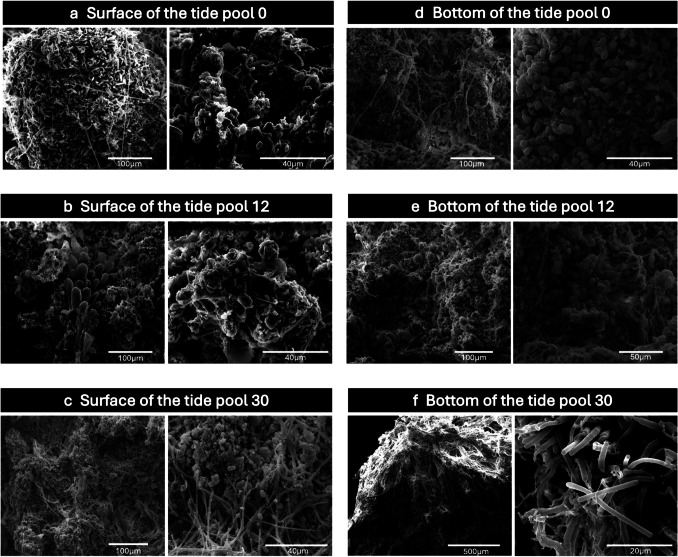
SEM of peritidal zone modern microbialites in three tide pools. **a**, **b**, **c** are surface microbialites in three pools, and **d**, **e**, **f** are bottom microbialites in three pools

SEM analysis revealed distinct morphological variations among the microbialites from the three examined tide pools (Fig. 2). Moreover, morphological differences between the surface and bottom microbialites within each tide pool were also observed, with the most pronounced disparity occurring in tide pool 30. Specifically, while the microbialite structures on the surface and bottom of tide pools 0, 12, and the surface of tide pool 30 exhibited more complex architectures, the microbialite structure at the bottom of tide pool 30 was notably simpler, with many long rod cells. The morphology of these long rod or filamentous cells is similar to the morphology of some members of *Myxococcus* genus and *Chloroflexota*. These observations suggest that the microbialite formations at the bottom of tide pool 30 were distinct from those found in other locations. We acknowledge that the absence of elemental analysis in this study represents a limitation. We recommend that future research incorporate elemental analysis and EDX punctual analyses to better elucidate the type of carbonate observed.

### Influence of Environmental Factors on Bacterial Alpha Diversity and Community Composition in Tide Pools

**Fig. 3:**
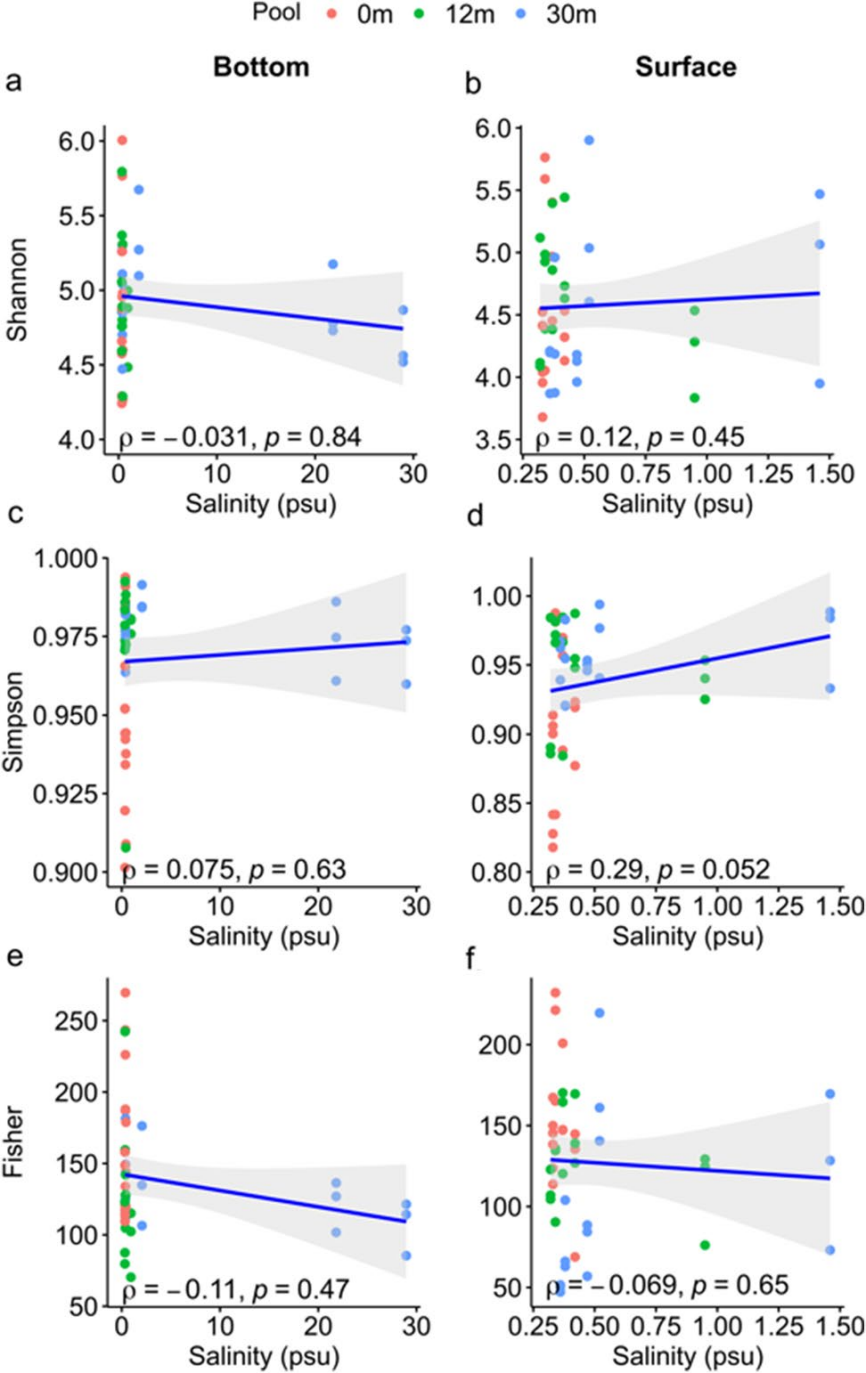
Correlation between salinity and bacterial alpha diversity of peritidal zone modern microbialites in three tide pools. **a** and **b** illustrate the correlation between salinity and bacterial Shannon diversity index at bottom and surface of pools, respectively. **c** and **d** depict the correlation between salinity and bacterial Simpson diversity index at bottom and surface, respectively. **e** and **f** show the correlation between salinity and bacterial Fisher diversity index at bottom and surface, respectively. The shaded areas in all panels represent the 95% confidence interval

The correlation between salinity and bacterial alpha diversity in microbialites from the three tide pools indicated that the disparity between the surface and bottom of tide pool 30 was more pronounced than in the other two tide pools (Fig. 3). Specifically, the salinity at the bottom of tide pool 30 was the highest, while it was comparatively lower in tide pools 0 and 12 (Fig. 3a, c, e). At the surface, the salinity of tide pool 30 was similar to that of tide pools 0 and 12 (Fig. 3b, d, f). However, there was no significant correlation between bacterial alpha diversity indices and salinity across the tide pools. These findings suggest that, while salinity differences highlight the distinct nature of the bottom environment in tide pool 30, bacterial alpha diversity does not reflect these differences.

Regarding temperature, there was minimal variation between the surface and bottom across the three tide pools (Fig. S1a, f, k). Among the alpha diversity indices, only the Shannon and Simpson indices at the bottom showed significant negative correlations with temperature (Shannon: *ρ* = − 0.37, *p* = 0.013; Simpson: *ρ* = − 0.30, *p* = 0.043; Fig. S1a, f). DO levels were consistently higher at both the surface and bottom of tide pool 30 compared to the other two pools (Tabel S1). However, only the Fisher index at the bottom showed a significant negative correlation with DO (*ρ* = − 0.39, *p* = 0.0074; Fig. S1l). TDS and SPC exhibited significant differences between its surface and bottom, particularly at the bottom, compared to the other tide pools (Fig. S1c, h, m, d, i, n). The pH levels were higher in tide pool 30, while tide pool 12 demonstrated the greatest variability (Fig. S1e, j, o). The Simpson index showed a significant positive correlation with pH at both the surface (*ρ* = 0.33, *p* = 0.026) and bottom (*ρ* = 0.39, *p* = 0.0076) (Fig. S1j). These results, combined with the earlier salinity findings (Fig. 3), indicate that tide pool 30 showed the most pronounced differences between its surface and bottom environments, with the bottom characterized by higher salinity, TDS, SPC, and pH.

CCA comparing the relationships between bacterial communities and environmental factors in the tide pools over time (Fig. S2) revealed that the bacterial communities in tide pool 30 showed a more significant correlation with environmental factors than those in the other tide pools. Specifically, in the January and October bottom samples of tide pool 30, bacterial composition was positively correlated with salinity, TDS, PSU, DO, and pH. In June, bacterial composition was positively correlated with pH. Overall, although only temperature, DO, and pH showed influence on bacterial alpha diversity, and the CCA results suggest that bacterial community composition was significantly influenced by more environmental factors, and pH was one of the most essential factors.

Regarding eukaryotic communities, the communities in tide pool 30 showed a more significant correlation with environmental factors than those in the other tide pools (Fig. S3), which was similar to the situation of bacterial communities. Among the environmental factors, pH was also an important factor to the eukaryotic communities, especially in March, June, and August.

### Eukaryotic and Prokaryotic Composition Variations in Tide Pools

**Fig. 4:**
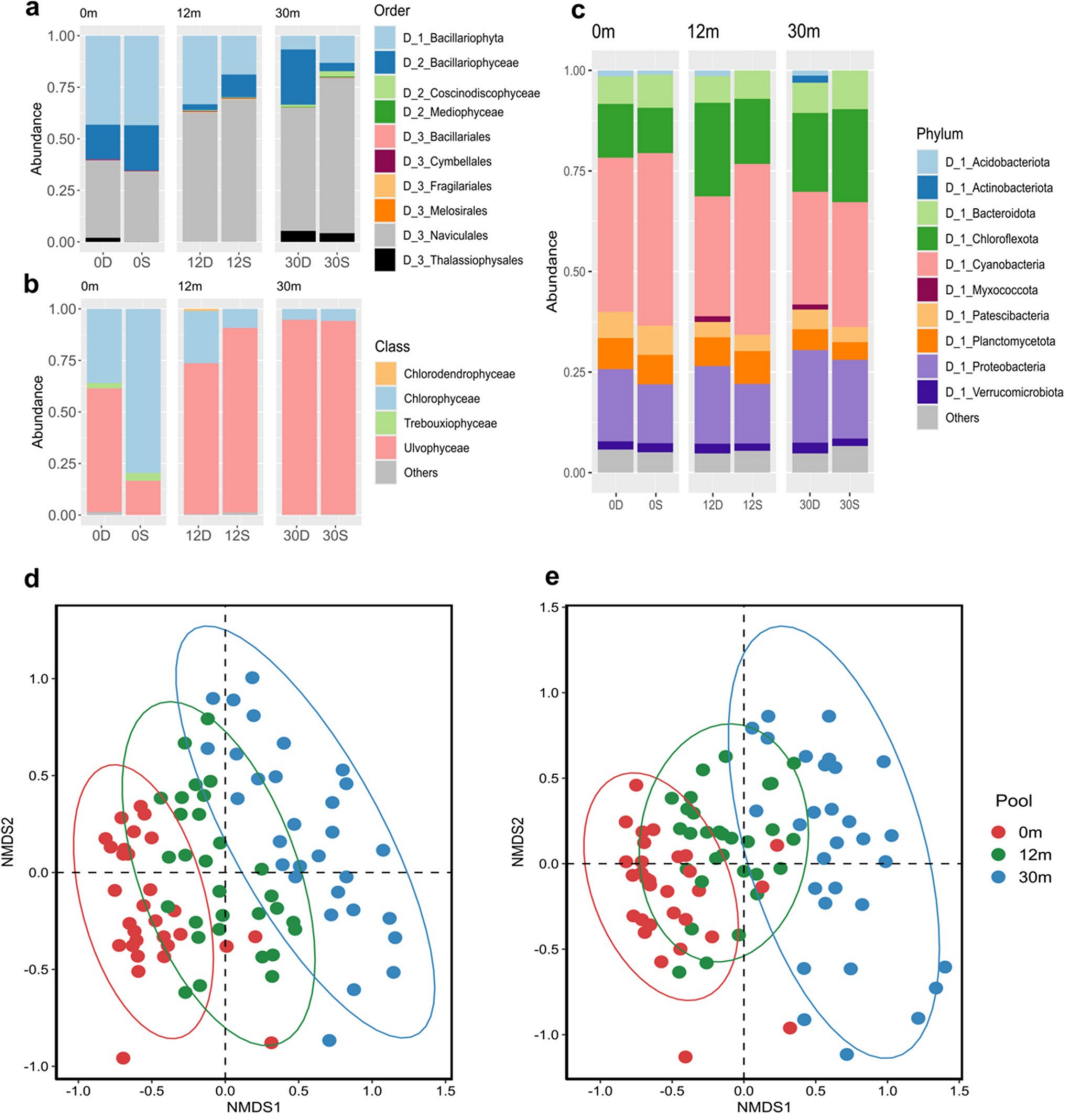
Bacterial and eukaryotic microorganism compositions and beta diversities in peritidal zone modern microbialites of tide pools. **a**–**b** Eukaryotic microorganism compositions of order level in diatom and class level in Chlorophyta, respectively. **c** Bacterial compositions of phylum level. **d** NMDS of the eukaryotic communities (stress: 0.2, PERMANOVA *p* = 0.001). **e** NMDS of the bacterial communities (stress: 0.15, PERMANOVA *p* = 0.001)

The analysis of eukaryotic composition in modern microbialites from the three tide pools (Fig. 4) indicated that Bacillariophyta were the predominant group within Ochrophyta, regardless of their proximity to seawater. Within Bacillariophyta (Fig. 4a), the class Bacillariophyceae, particularly the orders Naviculales and Thalassiophysales, was identified as the dominant group. An increased relative abundance of Naviculales was observed in tide pool 30. Additionally, the class Conscinodiscophyceae showed increased abundance in environments with higher seawater concentrations. In the Chlorophyta group (Fig. 4b), Ulvophyceae were more dominant in areas closer to seawater, while Chlorodendrophyceae and Trebouxiophyceae were detected only in environments with higher freshwater content. In the NMDS of eukaryotic composition (Fig. 4d), the eukaryotic KTUs in three tide pools were significantly different (stress: 0.2, PERMANOVA *p* = 0.001), which indicates that eukaryotic organisms were significantly different across the tide pools.

Regarding bacterial communities, the main bacterial composition of modern microbialites in the peritidal zones was consistent at the phylum level, with Cyanobacteria, Chloroflexota, and Proteobacteria being the most abundant (Fig. 4c). Notably, Myxococcota appeared at the bottom of tide pools 12 and 30, while Actinobacteriota were more prevalent at the bottom of tide pool 30. At the class level (Fig. S4), the differences in bacterial composition between the surface and bottom layers were more pronounced in areas closer to the seaside. Specifically, within the surface bacterial community of tide pool 30, Chloroflexia exhibited a higher relative abundance compared to the surface layers of tide pools 0 and 12. In the NMDS of bacterial composition (Fig. 4e), the bacterial KTUs in three tide pools showed a significant difference (stress: 0.15, PERMANOVA *p* = 0.001), which echoes the NMDS result of eukaryotic organisms.

**Fig. 5:**
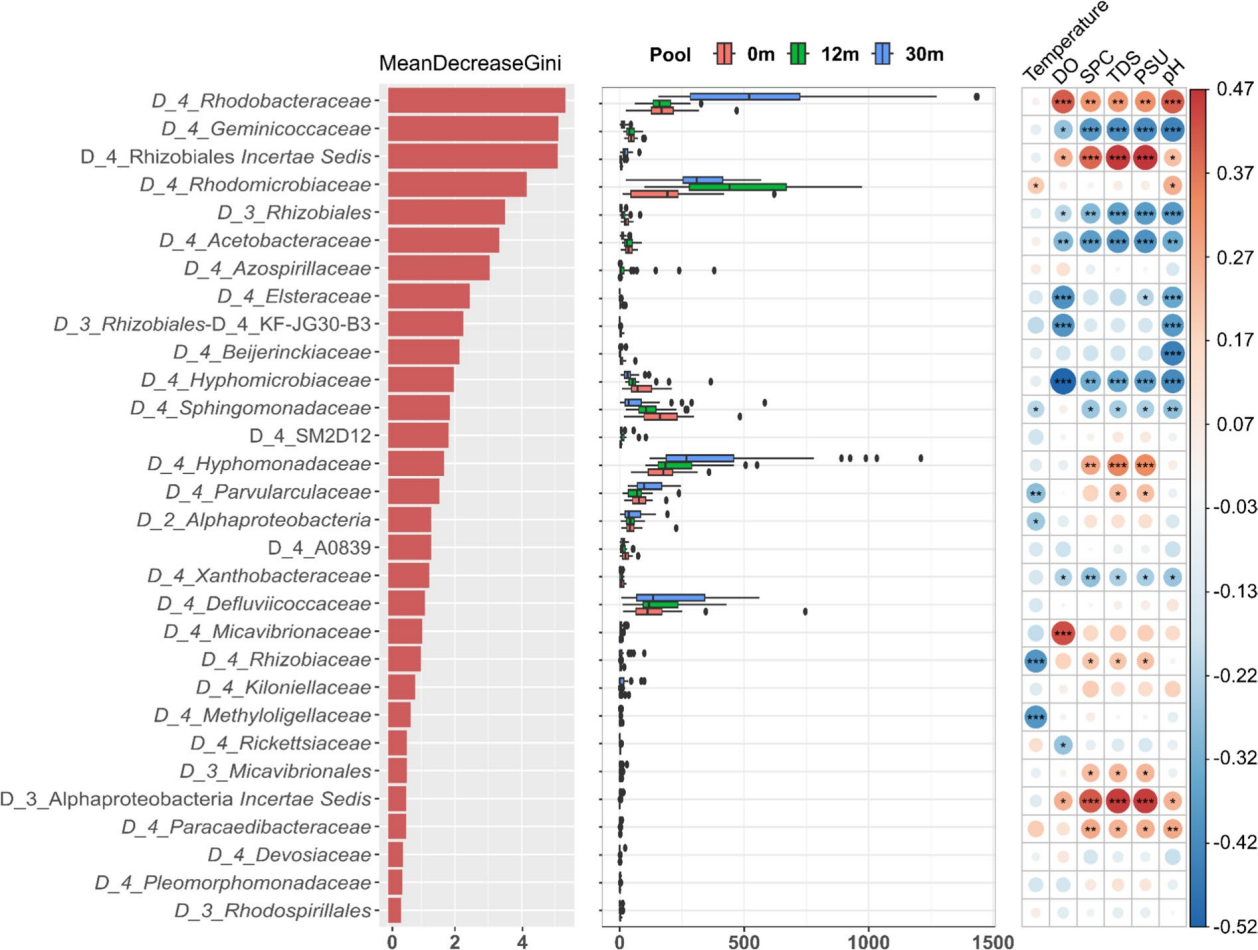
Random forest classification for Alphaproteobacteria and Spearman correlation with environmental factors. The left panel shows the random forest analysis results, identifying the most important families within Alphaproteobacteria across different tide pools. The middle panel presents the read counts of these families, while the right panel illustrates the Spearman rank correlation between read counts and environmental parameters (*p < 0.05; **p < 0.01; ***p < 0.001)

Within the Proteobacteria phylum (synonym Pseudomonadota), Alphaproteobacteria was identified as the most dominant class. The composition of different orders within Alphaproteobacteria varied according to the specific tide pool and the sampling time (Fig. S5). In tide pool 30, where salinity levels were the highest, the relative abundance of *Rhodobacterales* increased; *Rhizobiales* exhibited the highest relative abundance in tide pool 12. In tide pool 0, *Sphingomonadales* displayed a higher relative abundance compared to the other tide pools. To be more specific, according to the random forest analysis, which was performed to investigate the most important family in distinguishing pools, *Rhodobacteraceae* (*Rhodobacterales*), *Geminicoccaceae* (*Rhodospirillales*), two families of *Rhizobiales*, *Rhodomicrobiaceae* (*Hyphomicrobiales*), *Acetobacteraceae* (*Acetobacterales*), and *Azospirillaceae* (*Rhodospirillales*) were the important families with Gini values larger than 3, and their abundances were different in the three pools (Fig. 5). Spearman correlation revealed that the top 30 important families were significantly correlated (*p* < 0.05) with environmental factors. Among the factors, pH had more significant correlations with families with higher Gini values. Among the families with Gini values larger than 3, *Rhodobacteraceae* (*Rhodobacterales*) and Rhizobiales *incertae sedis*, which had higher abundances in tide pool 30 than other pools, showed significantly positive correlations with pH, PSU, TDS, SPC, and DO; *Rhodomicrobiaceae* (*Hyphomicrobiales*), which had higher abundances in the tide pool 12, only showed significantly positive correlations with pH and temperature; while *Geminicoccaceae* (*Rhodospirillales*), a family of *Rhizobiales* and *Acetobacteraceae* (*Acetobacterales*) showed significantly negative correlations with pH, PSU, TDS, SPC, and DO. The results indicate that different tide pools had different alphaproteobacterial taxa which were influenced by environmental factors, especially pH.

**Fig. 6:**
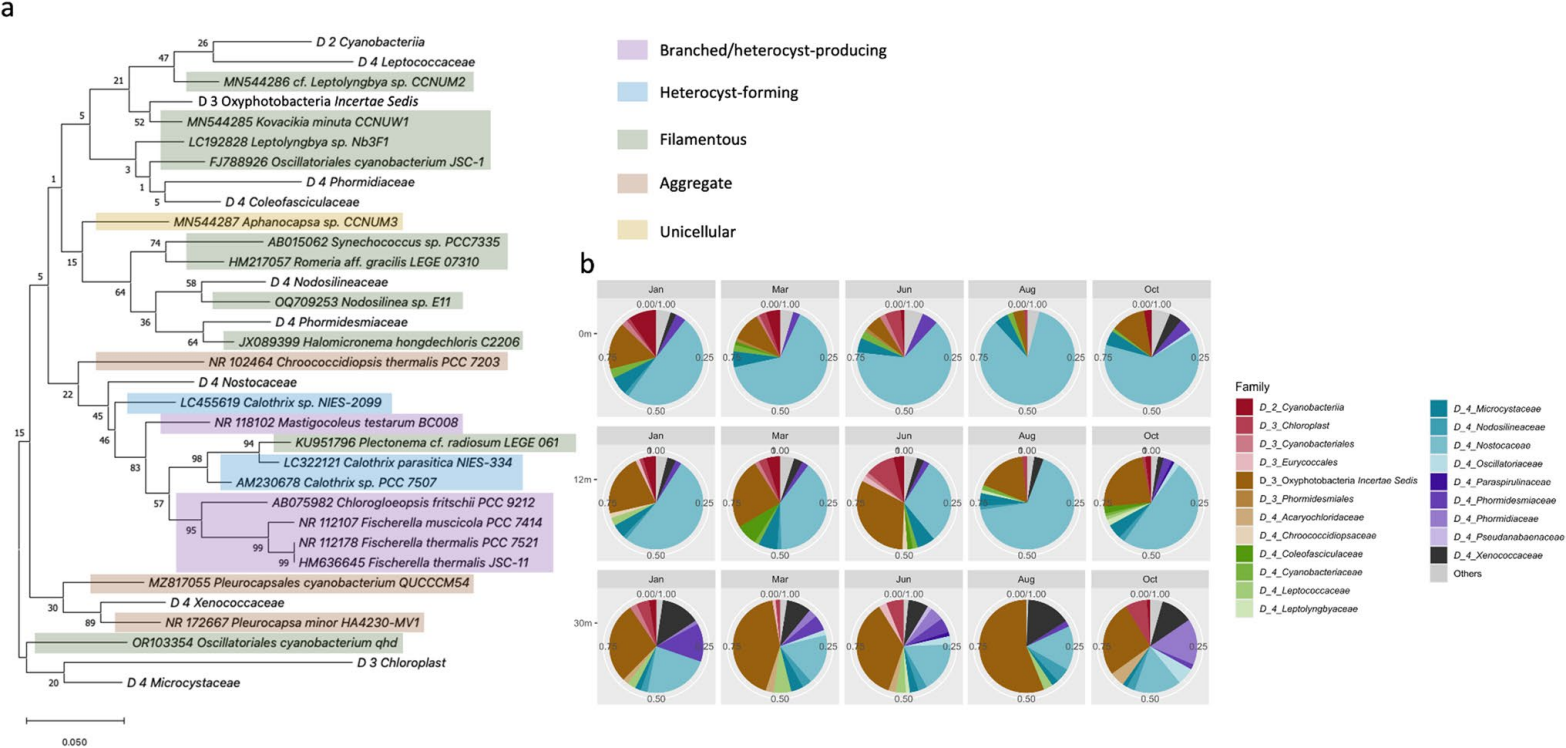
Phylogenetic tree and dynamics of cyanobacterial community in the peritidal zone modern microbialites during five sampling times. **a** In the phylogenetic tree, the different morphologies of Cyanobacteria are labelled with different colors. **b** The changes in the populations within the Cyanobacteria of modern microbialites in different tide pools across five different months

The composition of different families within Cyanobacteria varied across the tide pools. At different time points in tide pool 0, the family *Nostocaceae* was the dominant group. *Nostocaceae* is a family of heterocyst-forming Cyanobacteria capable of nitrogen fixation. In tide pool 12, both *Nostocaceae* and Oxyphotobacteria *incertae sedis* were dominant groups, with their relative abundances fluctuating seasonally (Fig. 6b). Phylogenetically, Oxyphotobacteria *incertae sedis* is closer to the filamentous families within *Synechococcales*, suggesting that this family consists of filamentous Cyanobacteria without heterocysts (Fig. 6a). In tide pool 30, Oxyphotobacteria *incertae sedis* generally exhibited higher relative abundance than *Nostocaceae*, and *Xanococcaceae* had increasing relative abundance. These findings indicate that in the peritidal zone, the dominant Cyanobacteria were primarily filamentous, and proximity to seawater was associated with a decrease in the relative abundance of heterocyst-containing Cyanobacteria.

Within the *Nostocaceae* family, there were clear differences in composition between the surface and bottom layers, particularly in tide pool 12 (Fig. S6), where *Rivularia* predominantly composed the bottom layer. *Rivularia* was also the main genus of *Nostocaceae* in tide pool 30. Compared to the other two tide pools, tide pool 12 exhibited more pronounced seasonal differences in the composition of the *Nostocaceae* family, resembling tide pool 30 more in winter and tide pool 0 more in summer (Fig. S6). This finding suggests that, despite the Cyanobacteria group being the most dominant in each tide pool, the composition of these communities was subject to change with environmental fluctuations.

According to the random forest analysis of cyanobacterial families (Fig. S7), *Cyanobacteriaceae*, *Microcystaceae*, *Xenococcaceae*, *Synechococcaceae*, *Nostocaceae*, Oxyphotobacteria *incertae sedis*, and *Nodosillneaceae* were the families with Gini values larger than 3, and their abundances were different in the three pools. Among them, *Xenococcaceae*, Oxyphotobacteria *incertae sedis*, and *Nodosillneaceae* had significantly positive Spearman correlation with pH (Spearman correlation, *p* < 0.001), others had significantly negative correlation with pH (Spearman correlation, *p* < 0.001). pH was the only factor that has a significant relationship with these seven families (Fig. S7).

With respect to Chloroflexia, which was also the one of dominant bacterial phyla in the samples, the divisions of SBR1031/*Aggregatilineales*, *Chloroflexaceae*, *Roseiflexaceae* and A4b were the important families with Gini values larger than 6 (Fig. S8). Among them, abundances of SBR1031/*Aggregatilineales*, *Chloroflexaceae*, and *Roseiflexaceae* were higher in tide pool 30 and had a significantly positive Spearman correlation with pH (Fig. S8, Spearman correlation, *p* < 0.01).

Focusing on bacterial communities in Myxococcota, which were predominant in the tide pool 30 or 12, pH, DO, and SPC were the factors that had significantly negative Spearman correlation with most of Myxococcota taxa, such as *Polyangiaceae* and *Haliangiaceae* (Spearman correlation, *p* < 0.001). Interestingly, the most dominant family, *Myxococcaceae*, only had a significant negative correlation with DO (Spearman correlation, *p* < 0.05), which may explain the higher abundance of Myxococcota at the bottoms of two tide pools (Fig. S9 and Fig. 4).

Similar to Myxococcota, Actinobacteriota was mainly found at the bottoms of tide pool 30. In the random forest analysis of Actinobacteriota (Fig. S10), the results showed that *Cellulomonadaceae* had Gini values exceeding 15, indicating that it was the most important family within Actinobacteriota. Moreover, the abundance of *Cellulomonadaceae* was significantly higher in tide pool 30 compared to other tide pools. Regarding its relationship with environmental parameters, *Cellulomonadaceae* exhibited significantly positive correlation with all environmental factors, particularly with pH (Spearman correlation, *p* < 0.01). The positive correlation between *Cellulomonadaceae* and PSU may explain its higher abundance in tide pool 30. Notably, *Cellulomonadaceae*’s relationship with DO seems not to provide an explanation for why it was more abundant at the bottoms of tide pools (Fig. S10 and Fig. 4). However, in the environmental parameters of tide pool 30 (Fig. S1), the DO range at the bottom was broader than that at the surface and can even exceed the surface DO values. This variation may explain why Actinobacteriota, dominated by *Cellulomonadaceae*, was more abundant at the bottom.

### Bacterial Network Associations with Dominant Cyanobacterial Genera

The bacterial network analysis revealed associations between bacterial genera and the dominant cyanobacterial genera based on their relative abundance. Since the previous results showed that the microbial community composition of the three tide pools exhibited gradient differences (Fig. 4), with the microbial composition in tide pool 12 serving as a transitional zone between the other two pools, the bacterial network analysis was conducted only for tide pool 0 and tide pool 30. In the bacterial network of tide pool 0 (Fig. S11a), *Nostocaceae* exhibited numerous connections with other bacteria (53 nodes), whereas Oxyphotobacteria *incertae sedis* showed fewer connections (24 nodes). Conversely, in the network of tide pool 30 (Fig. S11b), *Nostocaceae* had fewer connections with other bacteria (48 nodes), while Oxyphotobacteria *incertae sedis* displayed more extensive connections (55 nodes). These findings suggest that the bacterial composition within the microbialites may be influenced by the relative abundance of dominant cyanobacterial genera. Furthermore, the previously identified Chloroflexia (Chloroflexota) and *Myxococcaceae* (Myxococota) were also associated with the dominant Cyanobacteria, indicating potential interactions within these microbial communities.

### Functional Predictions of Bacterial Communities Across Tide Pools

**Fig. 7:**
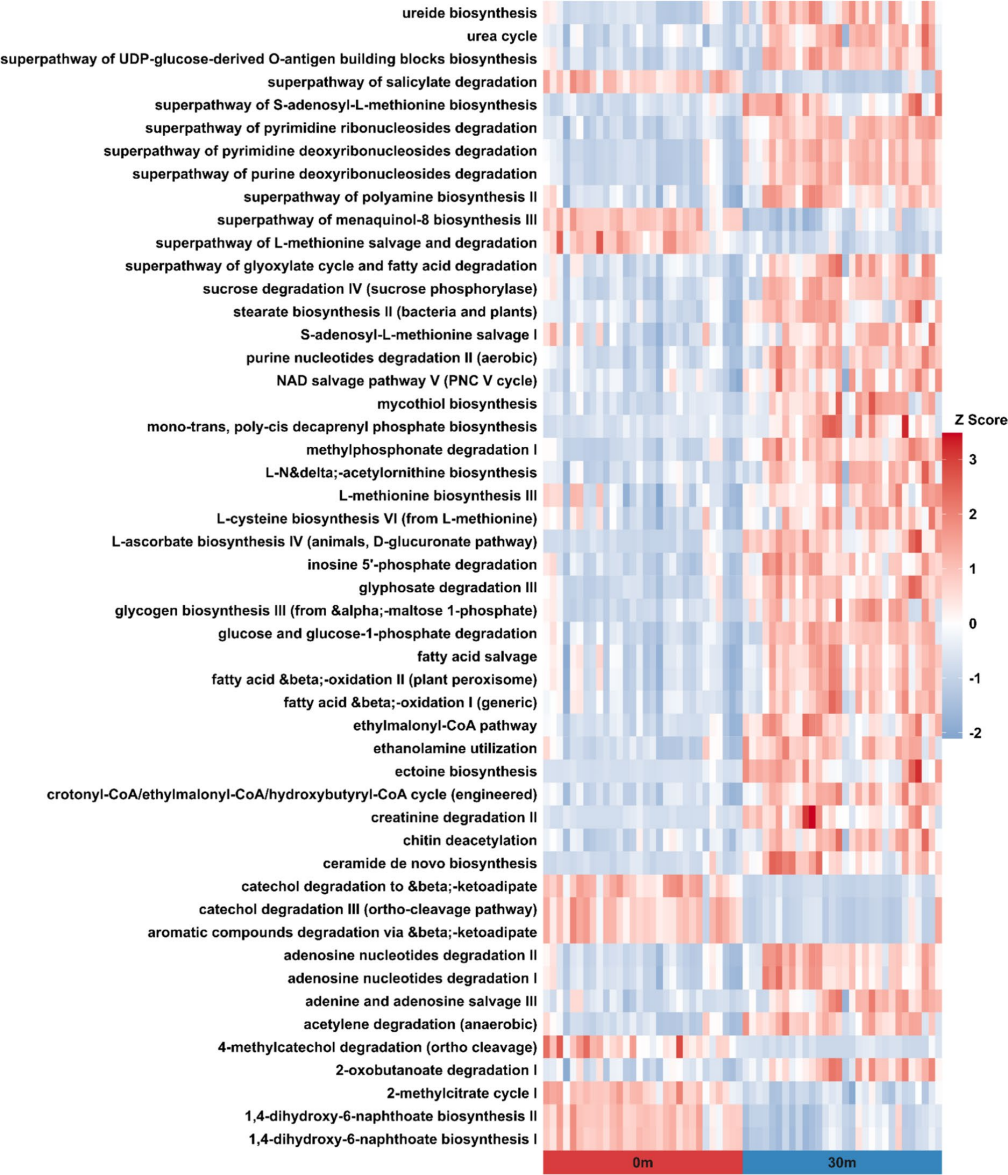
Top 50 differentially abundant functions between tide pools 0 and 30. Abundance values for each pathway were scaled using *z*-score normalization

To investigate functional changes in the bacterial communities, we employed PICRUSt2 to predict the potential functions of bacteria that exhibited significant differences between tide pools 0 and 30, as identified by LEfSe. The NMDS analysis revealed a clear theoretical metabolic functional shift from the land side (0 m) to the seaside (30 m) (Fig. S12, PERMANOVA *p* = 0.001). Among the top 50 predicted functions exhibiting the most significant differences between tide pools 0 and 30 (Fig. 7), the majority of metabolic pathways were enriched in tide pool 30. For instance, pathways associated with nucleic acid and amino acid metabolism, including adenosine nucleotide degradation, purine nucleotide degradation, and pyrimidine degradation, were more abundant in tide pool 30. Additionally, pathways related to the urea cycle were also enriched in tide pool 30.

## Discussion

The preservation of microbial mats in the fossil record is often constructed by carbonate precipitation, leading to the formation of lithified mats, also known as microbialites. These mats are vertically stratified, laminated communities shaped by gradients in light intensity, light quality, and oxygen availability. At the uppermost surface, Cyanobacteria and some members of the class Alphaproteobacteria, capable of oxygenic photosynthesis, are typically the dominant group [3, 20, 49]. As light and oxygen decrease with depth, the deeper layers become dominated by facultative anaerobic bacteria, including facultative anoxygenic phototrophs such as green non-sulfur bacteria (Chloroflexota) [49]. These phototrophic organisms are responsible for the majority of carbon dioxide fixation, and their metabolic byproducts, organic carbon exudates, serve as a nutrient source for heterotrophic bacteria. With further depletion of oxygen, strictly anaerobic phototrophs like green sulfur bacteria (Chlorobi) become dominant. These lower layers are usually black, sometimes interspersed with gray bands, reflecting high levels of sulfide and varying degrees of organic matter preservation [50]. In this study, the most dominant photoautotrophs were also Cyanobacteria, Chloroflexota, and predominantly photosynthetic Alphaproteobacteria, including members of *Rhodobacteraceae*, *Geminicoccaceae*, and *Rhodomicrobiaceae*, which is consistent with previous studies. Among these photoautotrophs, Cyanobacteria is known to play a central role in the formation of microbialite [16] and salinity significantly influences Cyanobacteria community composition [20, 51]. Same as this study, salinity was found to be one of the main factors influencing the bacteria composition. Additionally, our results further indicate that pH exhibits a higher correlation with Cyanobacteria and other photoautotrophs than salinity.

EPS plays a crucial role in the formation of microbialites. The high content of polysaccharides can trap and bind organisms and sediment for the base of microbialites; the surface is negatively charged, which can attract calcium ions to promote MICP; and all the metabolic activities can regulate local alkalinity which can favor MICP. Photoautotrophs Cyanobacteria produce EPS under environmental selective pressures, including variations in pH and salinity [52], and heterocytous cyanobacteria were identified as significant contributors to biofilm formation in most of the microbial mat types analyzed; among them, *Anabaena* spp. and *Nostoc* spp. (in the family *Nostocaceae*) are well-documented as key producers of EPS [53–55]. Heterocysts are specialized N-fixing cells, which is commonly found in freshwater environments due to adaptation for diazotrophic oxygenic photosynthetic growth under oxic conditions. The heterocystous cyanobacteria in this study, specifically members of the order *Nostocales*, were abundant in tide pool 0 and had a significantly negative correlation with salinity and pH (Spearman correlation, *p* < 0.001), while non-heterocystous, such as *Xenococcaceae* and Oxyphotobacteria *incertae sedis*, dominated at tide pool 30 with significantly positive correlation with salinity and pH (Spearman correlation, *p* < 0.001). These findings align with previous studies. Oren [56] proposed that salinity exerts a negative influence on the distribution of heterocystous cyanobacteria, particularly in normal marine to hypersaline environments. Campbell et al. [20] demonstrated that freshwater microbial mats in Australia favored heterocystous cyanobacteria under low salinity conditions, while higher salinities favored unicellular and filamentous non-heterocystous cyanobacterial genera. They further hypothesized that salinity could be controlling heterocyste glycolipid synthesis in Cyanobacteria in intertidal/subtidal zones. Our observations support this hypothesis. The change in the salinity level can clearly reflected not only in higher phylogenetic levels but also within the family level. For example, *Rivularia* was the main *Nostocaceae* genus in tide pools with higher salinity. In Shalygin et al.’s [57] study on the stromatolites of hypersaline lakes, *Rivularia* was found to be the dominant genus, indicating that *Rivularia* is likely the more salt-tolerant genus within the *Nostocaceae*. In this study, the genera within the family *Nostocaceae* show different compositions based on the salinity of the tide pool.

Other than Cyanobacteria, salinity and pH are also highly associated with other photoautotrophs in Alphaproteobacteria and Chloroflexota from this study. Within Alphaproteobacteria, *Rhodobacteraceae* and *Rhodomicrobiaceae* tend to exhibit a positive correlation with these environmental factors. In contrast to these photoautotrophs, diazotrophs within Alphaproteobacteria, such as *Rhizobiales*, showed a negative correlation with both pH and salinity. Regarding members in Chloroflexota, although certain Chloroflexota members are known to perform N-fixation [58], our results predominantly showed a positive correlation between salinity and the presence of phototrophs *Chloroflexaceae* and *Roseiflexaceae*, both of which lack N-fixing capabilities. Variations in the proportion of N-fixing bacteria have been observed across different tide pool environments. The high-energy demand of maintaining osmotic balance and expelling sodium ions while salinity increased [51], so the energy constraint may be a key factor in limiting nitrogenase activity and reducing fixation activity under high salinity [59]. Hence, the salinity and pH not only influence the composition of phototrophic communities but could also affect nitrogen cycling. The reduction of N-fixation might further lead to a shift in trophic strategy in higher salinity tide pools, which could favor alternative nitrogen metabolic processing like ammonification of amino acids and ureolysis, for the nitrogen source.

In microbial mats, biological metabolic activities can influence abiotic environmental factors, thereby affecting local alkalinity and the availability of free calcium ions, which in turn regulate carbonate precipitation [60]. Photosynthetic bacteria remove CO₂ from the environment during carbon fixation, this often occurs at a rate exceeding the replenishment of CO₂ via diffusion, leading to the dissociation of HCO₃⁻ into CO₂ and OH⁻. The resulting increase in alkalinity facilitates the precipitation of CaCO₃. In addition to photosynthesis, sulfate-reducing bacteria (SRB) in marine and hypersaline microbial mats play a critical role in shaping community metabolism, primarily by increasing alkalinity and thereby promoting calcium carbonate precipitation [60]. In this study, *Desulfobacteria* were found predominantly in tide pool 30, which is highly influenced by seawater.

Other microbial processes like heterotrophic metabolism, sulfide oxidation, and fermentation processes can lead to the decrease of alkalinity, leading to the dissolution of CaCO₃ [60]. However, a lower pH will degrade the labile fraction of the EPS, this can release Ca^2+^ previously bound to the polymers. Distinct heterotrophic community compositions were observed across different tide pools. Some of these heterotrophs are capable of degrading complex carbon sources or polysaccharides, which might lead to degraded EPS, for example, *Phycisphaerae* and *Chitinophagaceae*, which showed negative correlations with salinity and pH. In another hand, *Cyclobacteriaceae*, *Ignavibacteria*, *Myxococcia*, *Cellulomonadaceae*, and the SRB *Desulfobacteria*, exhibited positive correlations with salinity and pH.

Reactions and by-products involved in various microbial metabolic pathways can also lead to MICP. Apart from the feasibility MICP by photosynthesis of Cyanobacteria, ammonification of amino acids and urea hydrolysis may be one of the heterotrophic MICP factors in the microbialites. In the process of ammonification of amino acids, their metabolism produces CO₂ and ammonia. Additionally, N-fixation also generates ammonia. The hydrolysis of ammonia generates NH₄⁺ and OH⁻ around the cell, increasing local alkalinity and leading to supersaturation, which consequently promotes CaCO_3_ precipitation [16]. Many heterotrophic bacteria are capable of utilizing amino acids, including *Myxococcus xanthus*, a member of the *Myxococcaceae* family, commonly referred to as slime bacteria, which has been shown to act as nucleation templates for carbonate precipitation [61]. In addition to being aerobic and rod-shaped, they are characterized by their ability to utilize amino acids as their sole energy source [62, 63]. In our results, the relative abundance of *Myxococcaceae* increases in the bottoms of tide pools 12 and 30, and the rod-shaped cells which were similar to *Myxococcus* [64] can be observed in the bottoms of tide pool 30. Although the members of *Myxococcaceae* were not *M. xanthus*, it remains possible that these members contribute to CaCO₃ precipitation as a by-product of the ammonification process.

Ureolysis as part of ammonification involves bacterial urease hydrolyzing urea in the environment into CO_2_ and ammonia, with ammonia dissolving in water and dissociating into NH₄⁺ and OH⁻. This indirectly raises the microenvironment’s pH, promoting the precipitation of calcium ions as  CaCO_3_. The most optimal pH for urease activity is around pH 8, and activity decreases as pH level rises. In the study by Nguyen et al. [17] on microbialite-forming mats from South Australian saline lakes, the potential for microorganisms to participate in carbonate precipitation through ureolysis was proposed. The relatively high abundance of *Cellulomonadaceae* found at the bottoms of tide pool 30, where pH is between 8.09 to 8.35, may support Nguyen’s finding. Some genera in *Cellulomonadaceae* are known to produce urease [65]. *Cellulomonadaceae* had significantly positive correlations (Spearman correlation, *p* < 0.01) with all the environmental factors, especially pH. Therefore, they might have metabolic potential to break down urea via ureases and contribute to carbonate precipitation. Additionally, in tide pool 30, the relative abundance of *Rhodobacteraceae* (Alphaproteobacteria) and *Xenococcaceae* (Cyanobacteria) was higher. Similar to Nguyen et al.’s [17] findings, genes related to ureolysis were enriched in the microbialite-forming mats primarily from *Rhodobacteraceae* and *Xenococcaceae*. Based on the PICRUSt2 results, there are also a higher degree of confidence of urea cycle pathway in tide pool 30. Based on these findings, it suggests that microorganisms in tide pool 30 may contribute to CaCO₃ precipitation through ureolysis.

In the peritidal zone modern microbialites, diatoms are common eukaryotic microorganisms [3]. Diatoms possess a urea cycle [66]; although, it is uncertain whether the CO_2_ and ammonia produced from urea decomposition within their cells or directly dissolve in water, and diatoms may participate in the MICP process to some extent. Regarding the relationship between diatom and the dominant bacterial in the peritidal zone modern microbialites, previous studies have found that the diatoms *Hemiaulus*, *Rhizosolenia*, and *Chaetoceros* form symbiotic relationships with heterocystous cyanobacteria, such as *Richelia* and *Calothrix* (both are in the order *Nostocales*) [67, 68]. These three genera of diatoms belong to the class Bacillariophyceae. Diatoms predominantly belong to the class Bacillariophyceae in this study, with a relatively higher abundance in the less saline tide pool 0. Meanwhile, *Calothrix* is also the dominant Cyanobacteria in tide pool 0. A higher N-fixation activity in heterocystous cyanobacteria, including *Nostocales* [20, 51], can be found in lower salinity conditions. Although it is currently unclear whether there is a direct relationship between diatoms and Cyanobacteria in peritidal zone modern microbialites, it is speculated that nitrogen utilization may be involved in the composition of diatoms and taxonomic assemblage of cyanobacterial populations. In future studies on MICP in modern microbialites, it is recommended to consider the role of diatoms as a subject of research, and their relationships among Cyanobacteria and other microorganisms.

This study highlights the intricate interplay between microbial community composition, metabolic pathways, and environmental parameters in shaping microbialite formations. The vertical stratification of microbial mats reflects strong environmental gradients, where salinity and pH emerge as key factors influencing the distribution of photoautotrophs, particularly Cyanobacteria. Our results confirm that heterocystous cyanobacteria favor low salinity and pH conditions, while non-heterocystous genera dominate more marine-like environments. Beyond phototrophic composition, N-fixation appears to be suppressed under high salinity, potentially prompting a shift toward urea or amino acids utilization as an alternative N source. In addition to photosynthesis, heterotrophic metabolic processes, including ammonification of amino acids and ureolysis, also contribute to MICP by increasing local alkalinity through the release of NH₄⁺ and OH⁻ under high salinity and pH conditions. Specific heterotrophic taxa, such as *Myxococcaceae* and *Cellulomonadaceae*, may play an active role in these processes, especially in high pH and high salinity conditions such as tide pool 30. The elevated abundance of taxa associated with ureolysis, together with the inferred presence of the urea cycle, further supports the role of heterotrophic pathways in carbonate precipitation. These findings suggest that microbialite formation results from a complex network of metabolic interactions influenced by environmental pressures, emphasizing the need to consider both autotrophic and heterotrophic contributions to mineralization in modern microbial mats.

## Supplementary Information

Below is the link to the electronic supplementary material.

Supplementary file1 (PDF 1776 KB)

## Data Availability

The sequenced data could be found at NCBI under BioProject accession number: PRJNA1164468.
