# Supplementary material for: Microbial Community Shifts and Nitrogen Utilization in Peritidal Microbialites: The Role of Salinity and pH in Microbially Induced Carbonate Precipitation
Source: Microb Ecol. 2025 Apr 22;88:31. doi: 10.1007/s00248-025-02532-1 (PMC12011901; doi:10.1007/s00248-025-02532-1)
Supplement: Supplementary file 1 [file 248_2025_2532_MOESM1_ESM.pdf]

# Microbial Community Shifts and Nitrogen Utilization in Peritidal Microbialites: The Role of Salinity and pH in Microbially Induced Carbonate Precipitation

Yunli Eric Hsieh[1,2,3], Sung-Yin Yang[4], Shao-Lun Liu[5], Shih-Wei Wang[6], Wei-Lung Wang[7], Sen-Lin Tang[8], Shan-Hua Yang[9,#]

**Affiliations:**

[1]Systems Biology and Mathematical Modeling Group, Max Planck Institute of Molecular Plant Physiology, Potsdam, Germany

[2]Bioinformatics Department, Institute of Biochemistry and Biology, University of Potsdam, Potsdam, Germany

[3]School of BioSciences, The University of Melbourne, Parkville, Australia

[4]Department of Aquatic Bioscience, National Chiayi University, Chiayi, Taiwan

[5]Department of Life Science & Center for Ecology and Environment, Tunghai University, Taichung, Taiwan

[6]Department of Geology, National Museum of Natural Science, Taichung, Taiwan

[7]Department of Biology, National Changhua University of Education, Changhua, Taiwan

[8]Biodiversity Research Center, Academia Sinica, Taipei, Taiwan

[9]Institute of Fisheries Science, National Taiwan University, Taipei, Taiwan

* Corresponding author: Shan-Hua Yang, shanhua@ntu.edu.tw

**Supplementary material**

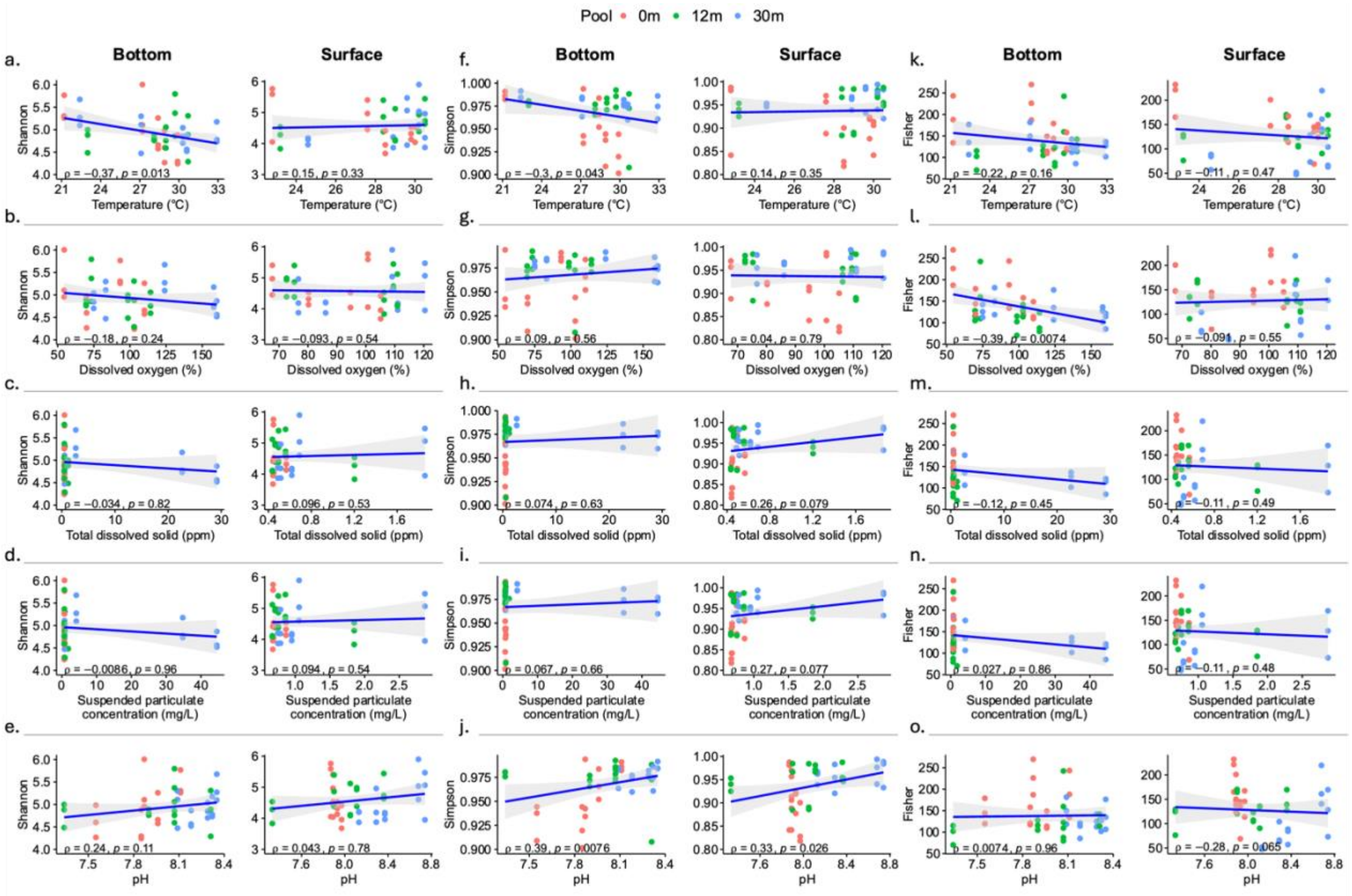


**Fig. S1 The correlation between environmental factors and bacterial alpha-diversity in bottom and surface of tide pools** Spearman correlation analysis was used to assess the relationships between alpha-diversity indices (Shannon, Simpson, and Fisher) and environmental factors, including temperature, dissolved oxygen, total dissolved

solids, suspended particulate concentration, and pH. The shaded areas in all panels represent the 95% confidence interval.

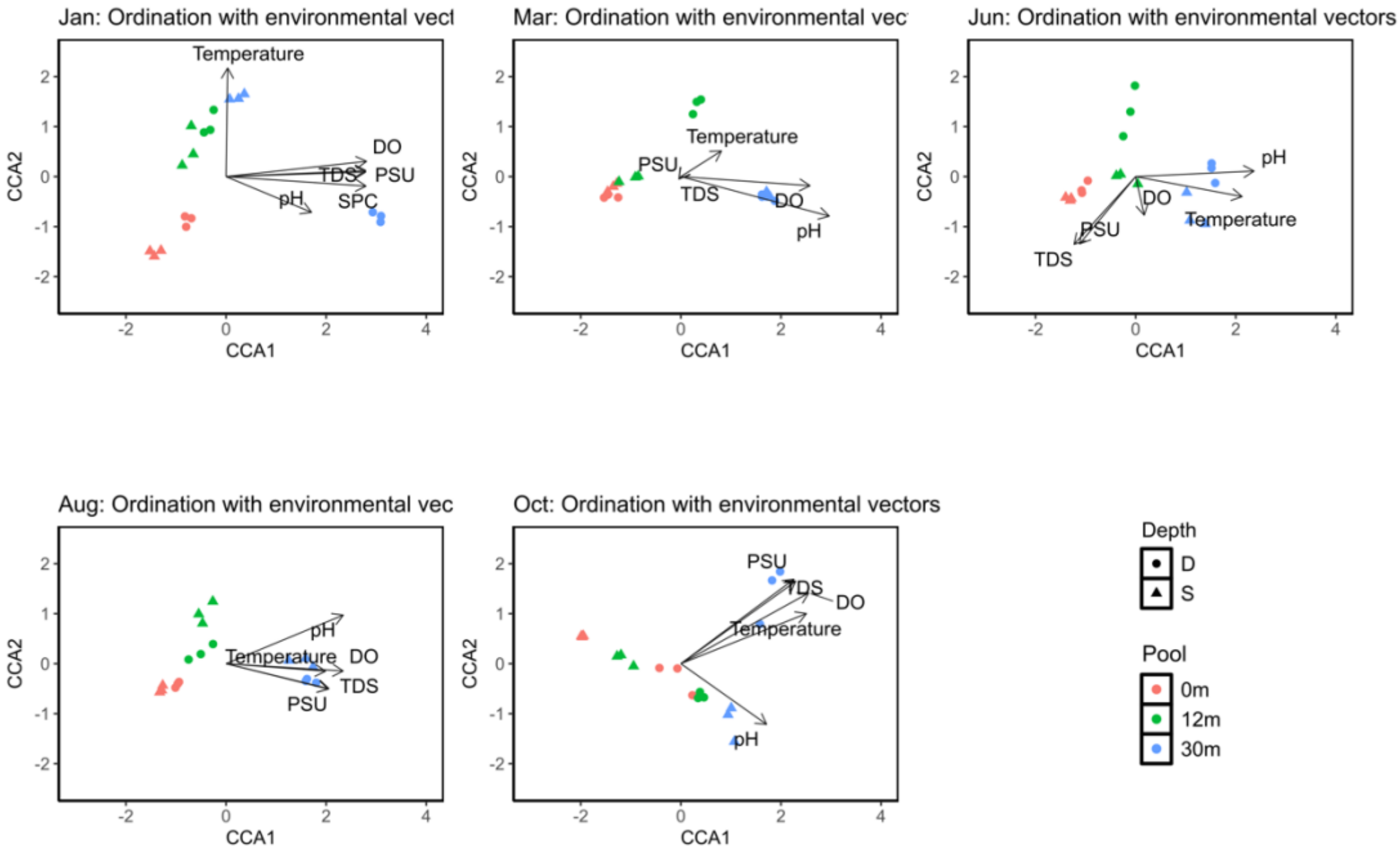


**Fig. S2 Canonical correspondence analysis (CCA) of bacterial communities of three tidal pools (0m, 12m, 30m)** D indicates the bottom part of the tidal pools, and S indicates the surface part of the tidal pools. The number of KTUs was 6863.

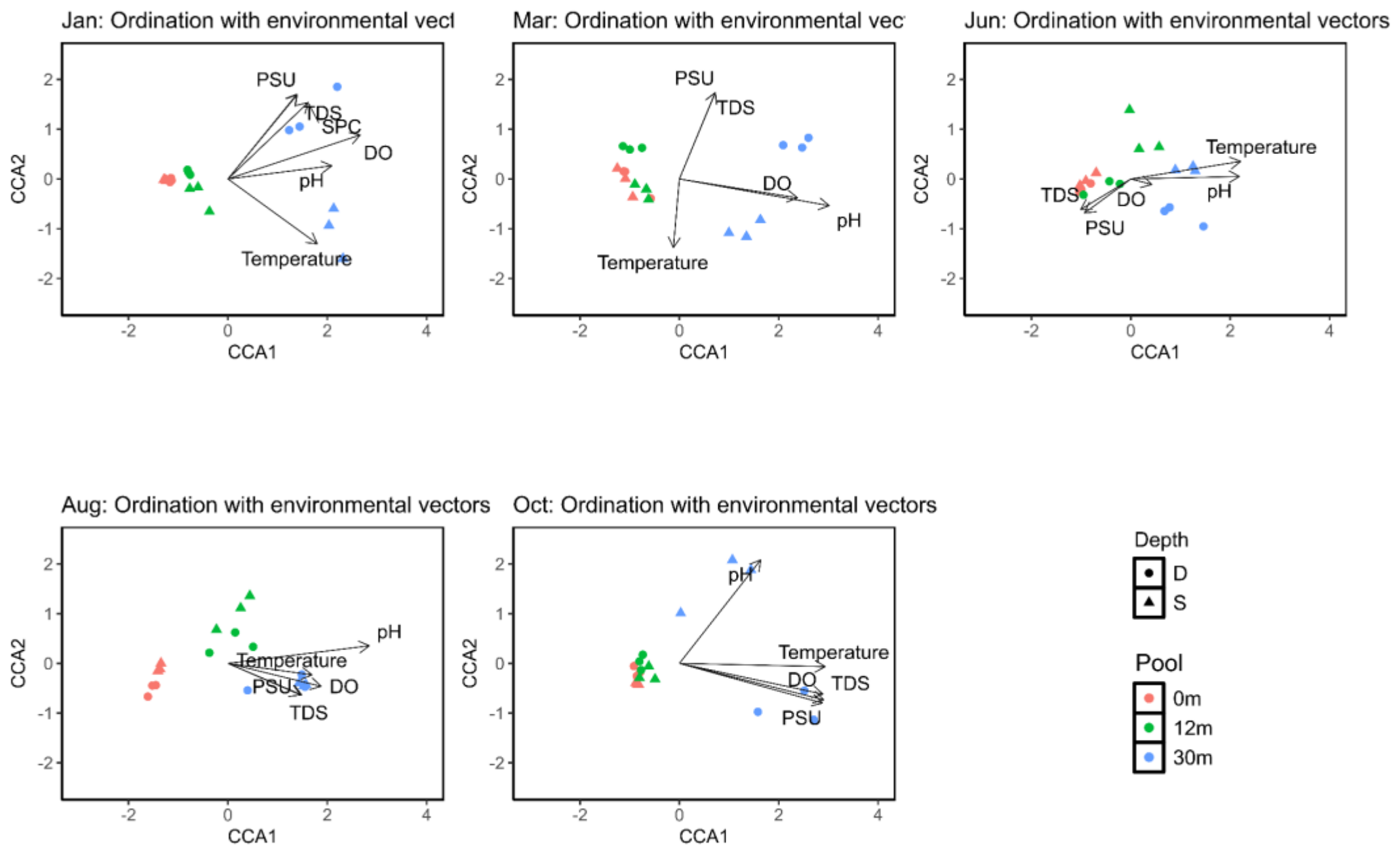


**Fig. S3 Canonical correspondence analysis (CCA) of eukaryotic communities of three tidal pools (0m, 12m, 30m)** D indicates the bottom part of the tidal pools, and S indicates the surface part of the tidal pools. The number of KTUs was 1724.

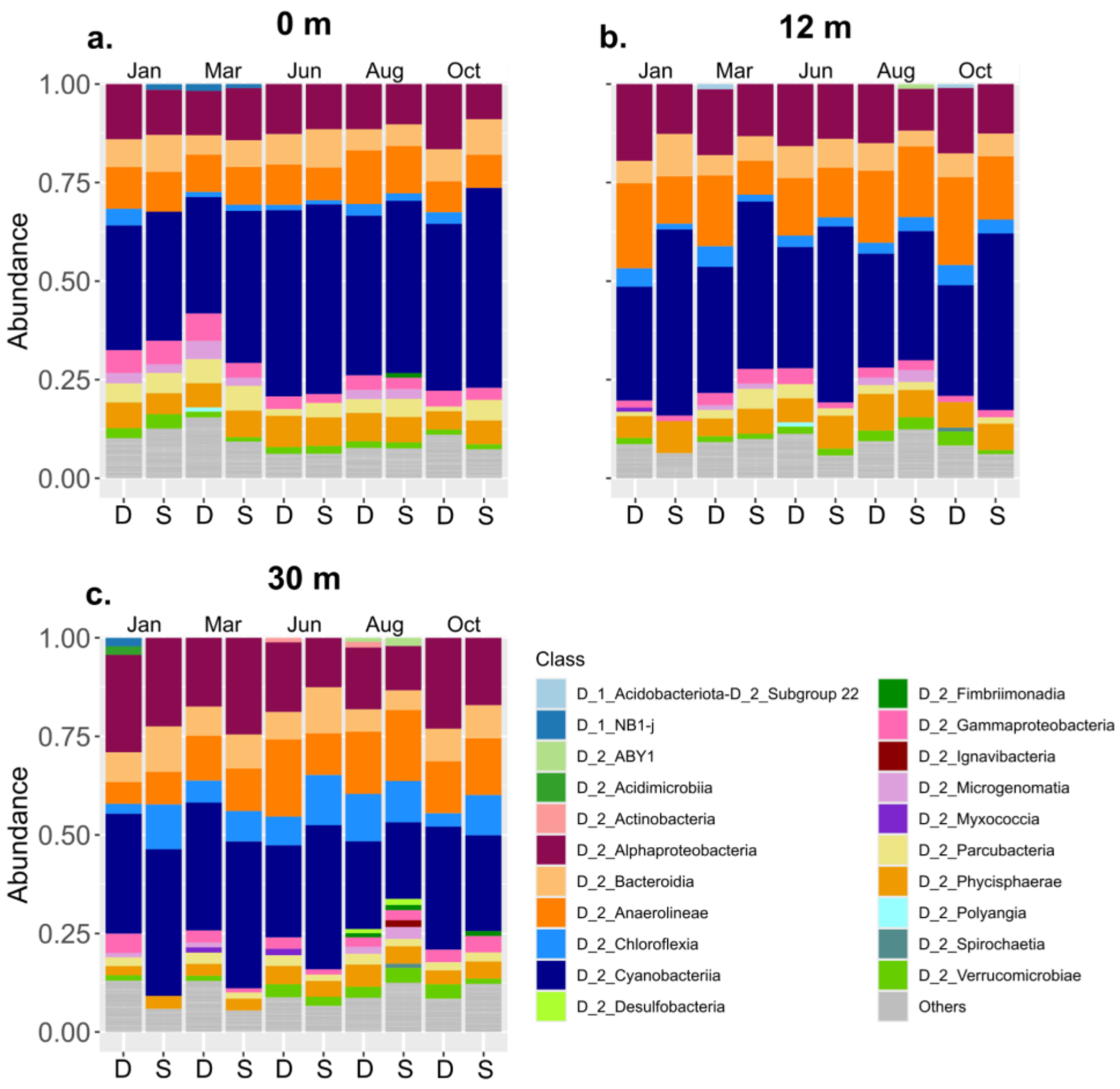


**Fig. S4 Bacterial compositions in modern microbialites of tide pools** Bacterial compositions at class level during five sampling times: (a) tide pool 0; (b) tide pool 12; (c) tide pool 30. S indicates the surface part of the pools, and D indicates the bottom part of the pools.

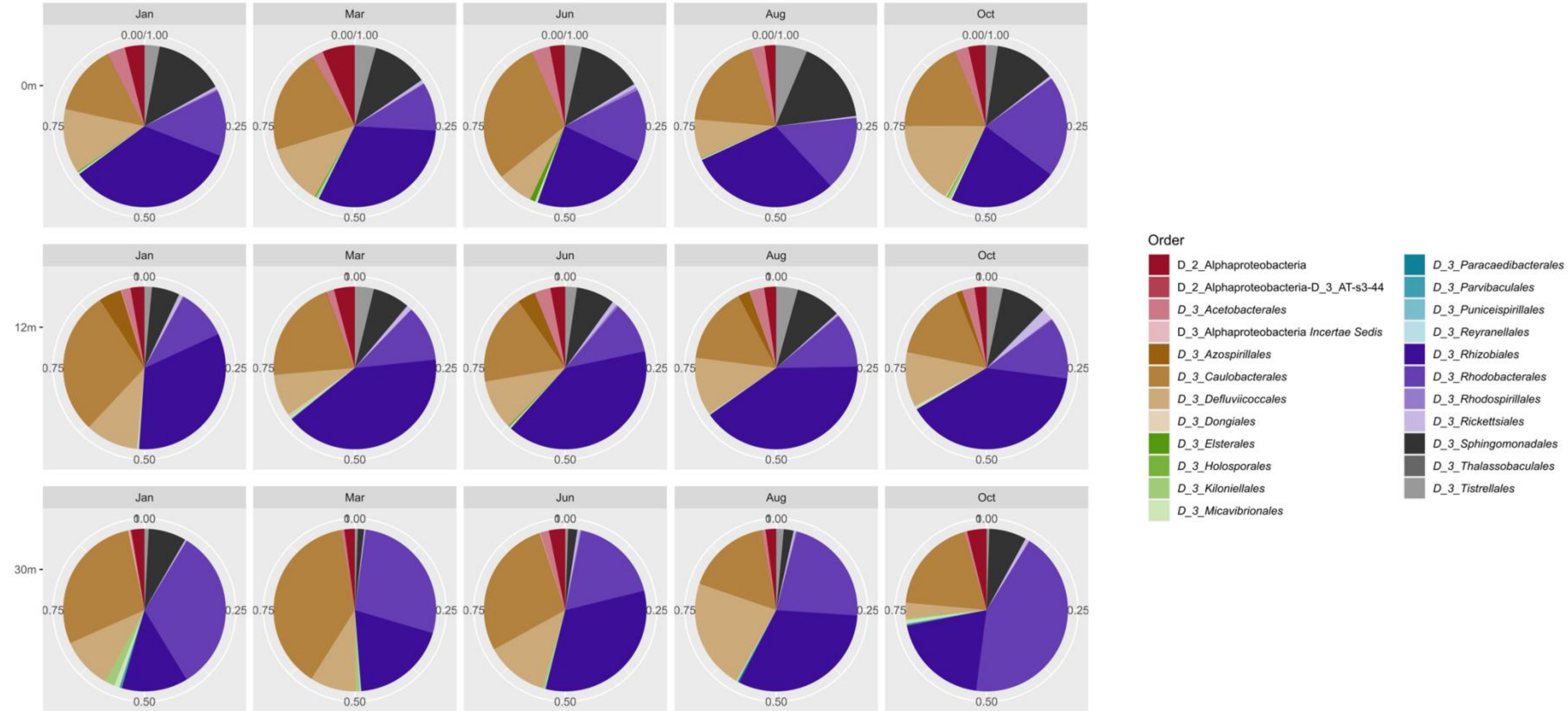


**Fig. S5 The changes in the populations within the Alphaproteobacteria of modern microbialites in different tide pools across five different months** Colors indicate different orders in the class.

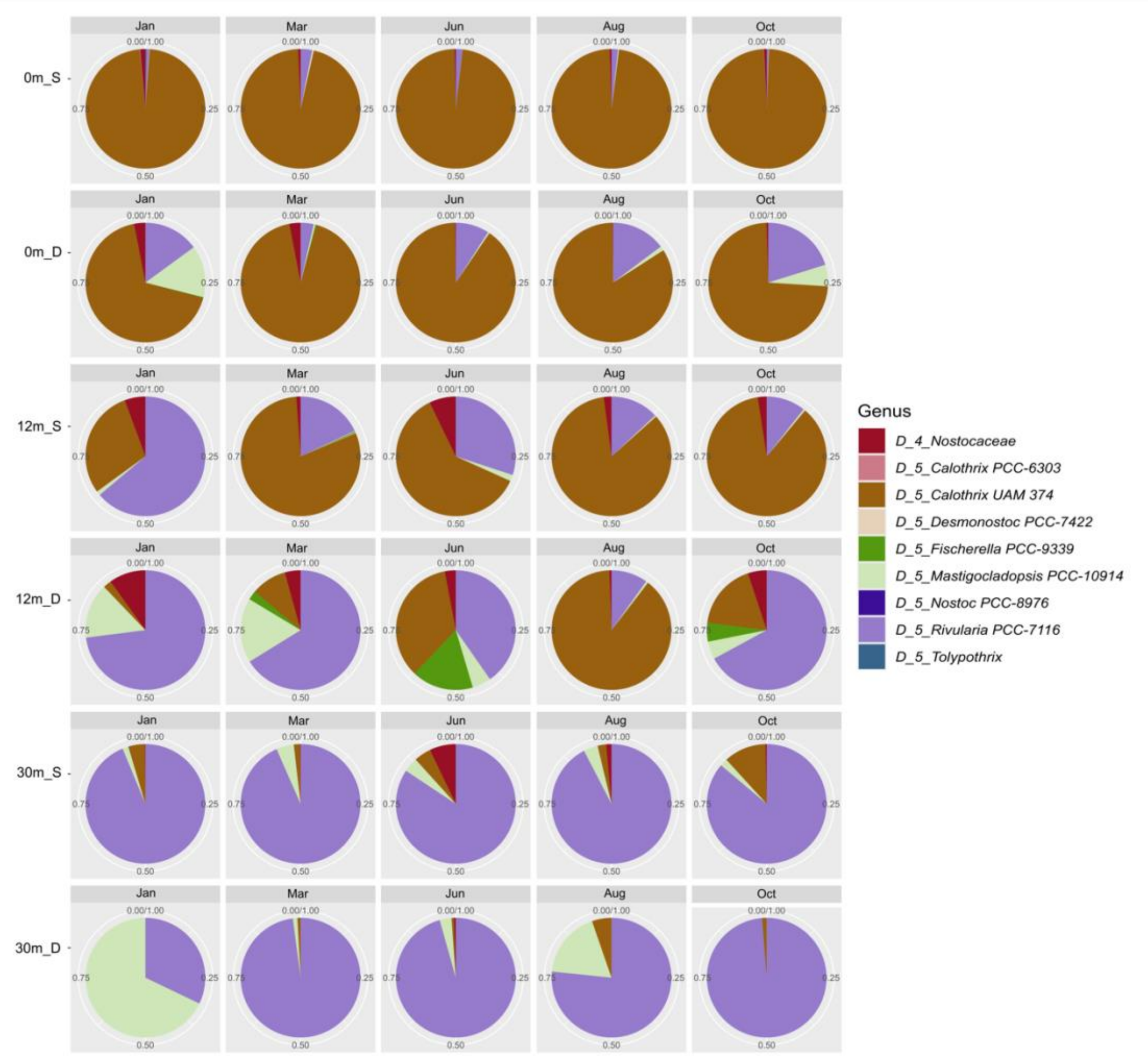


**Fig. S6 The changes in the populations within the *Nostocaceae* family of modern microbialites in different tide pools at five time points** Colors indicate different genera in the family.

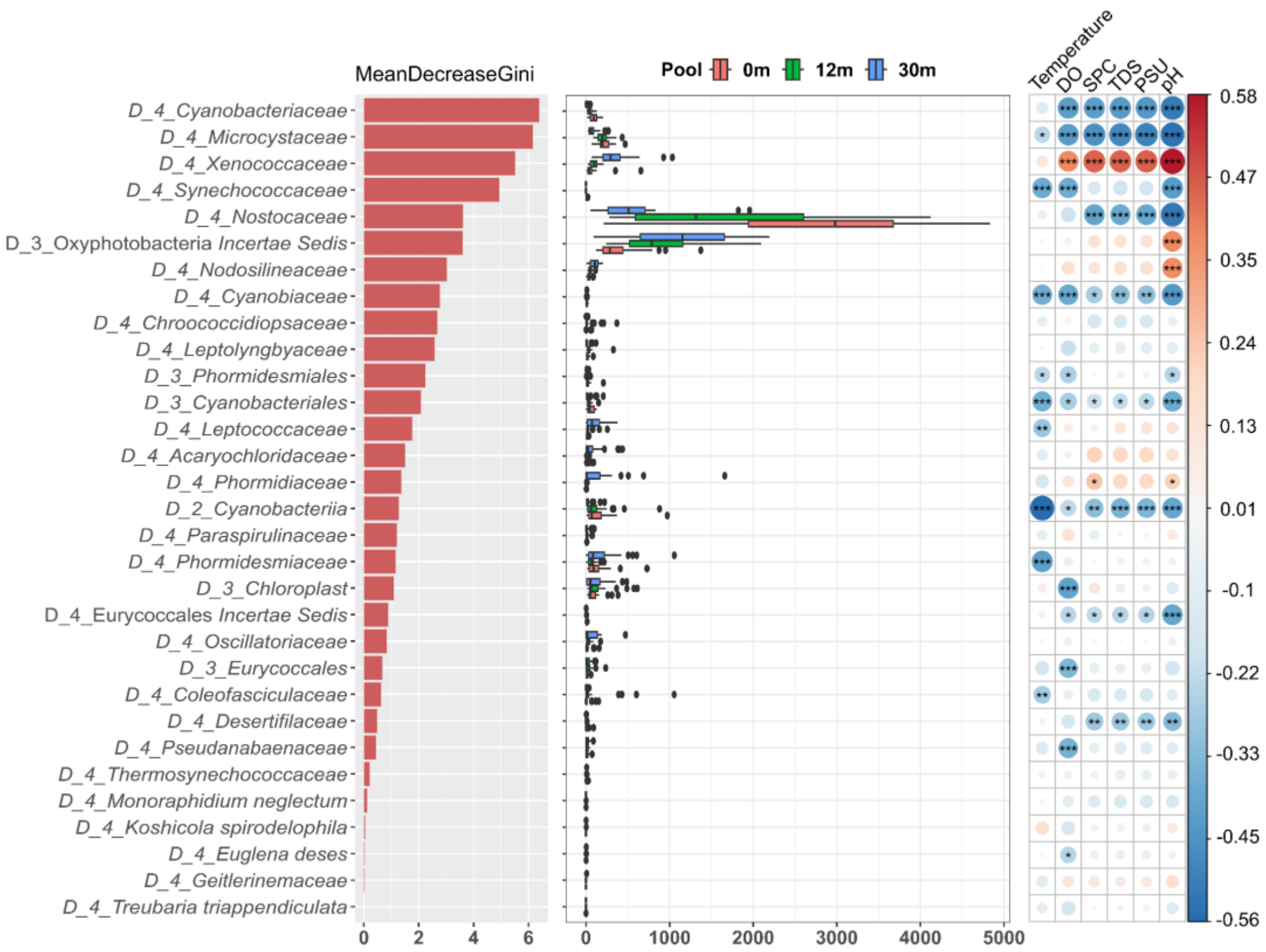


**Fig. S7 Random Forest classification for cyanobacterial families and Spearman correlation with environmental factors** The left panel shows the Random Forest analysis results, identifying the most important families within Cyanobacteria across different tide pools. The middle panel presents the read counts of these families, while the right panel illustrates the Spearman rank correlation between read counts and environmental parameters (* $p < 0.05$; ** $p < 0.01$; *** $p < 0.001$).

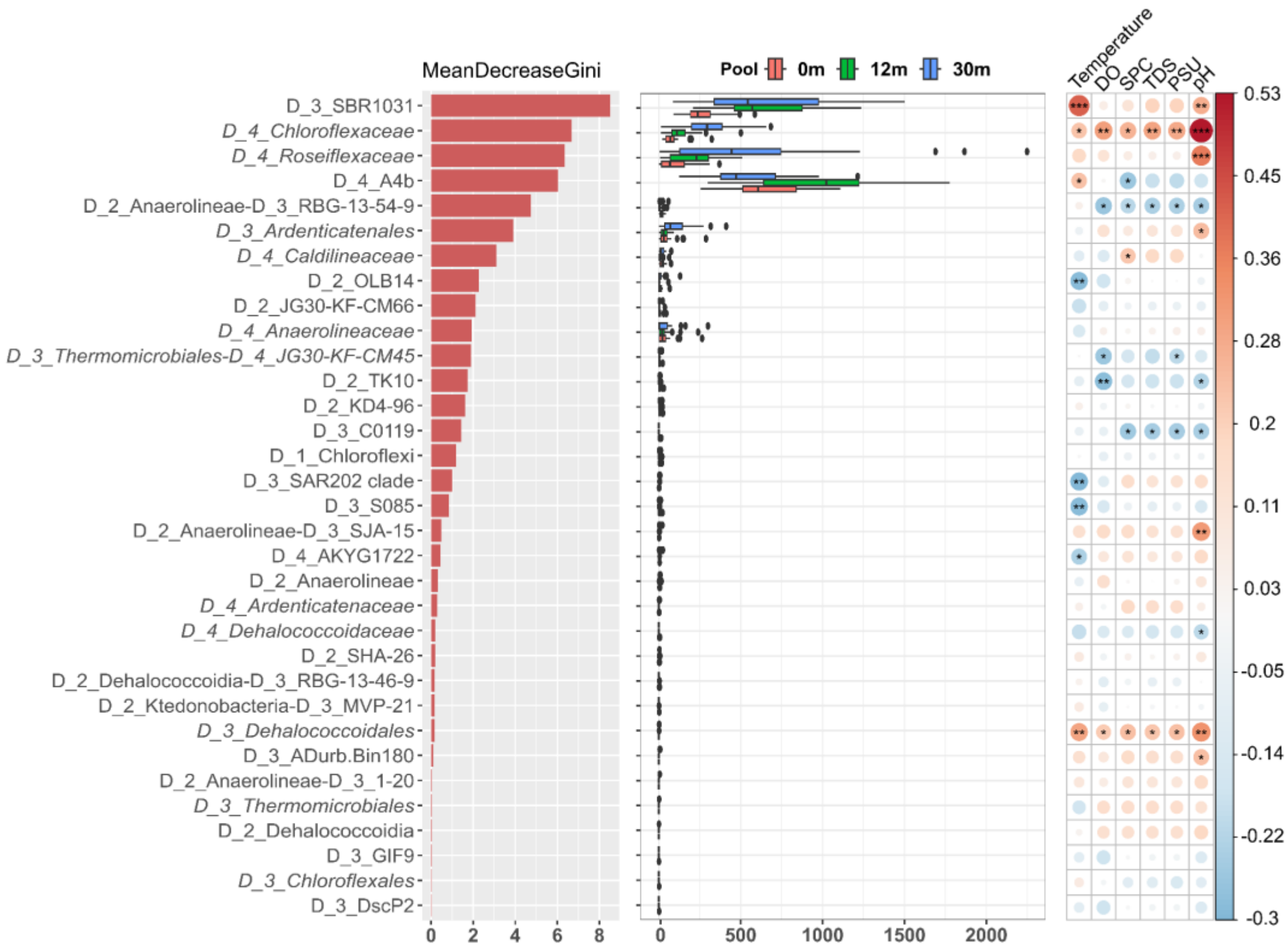


**Fig. S8 Random Forest classification for families of Chloroflexota and Spearman correlation with environmental factors** The left panel shows the Random Forest analysis results, identifying the most important families within Chloroflexota across different tide pools. The middle panel presents the read counts of these families, while the right panel illustrates the Spearman rank correlation between read counts and environmental parameters (* $p < 0.05$; ** $p < 0.01$; *** $p < 0.001$).

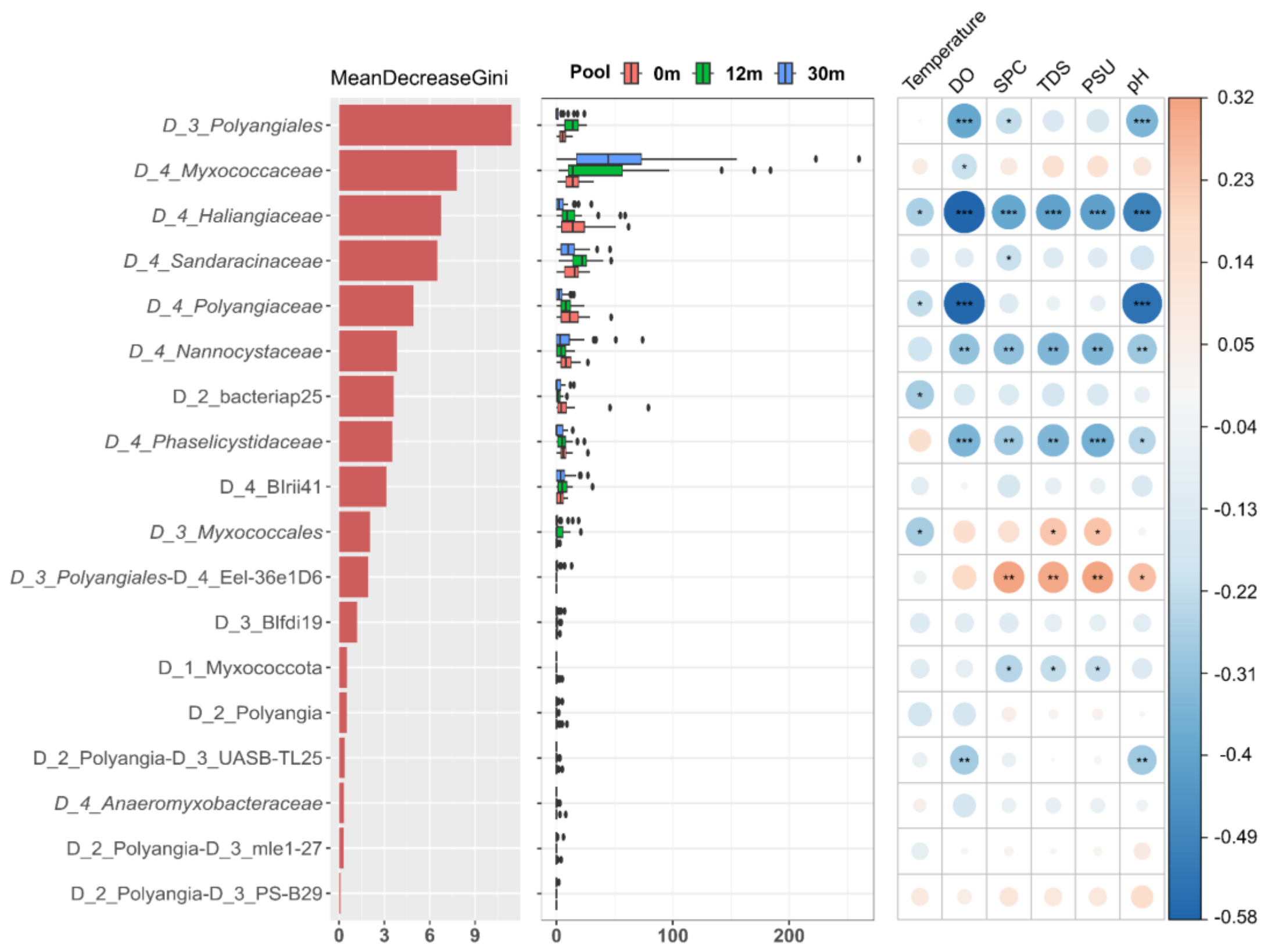


**Fig. S9 Random Forest classification for families of Myxococcota and Spearman correlation with environmental factors** The left panel shows the Random Forest analysis results, identifying the most important families within Myxococcota across different tide pools. The middle panel presents the read counts of these families, while the right panel illustrates the Spearman rank correlation between read counts and environmental parameters (* $p < 0.05$; ** $p < 0.01$; *** $p < 0.001$).

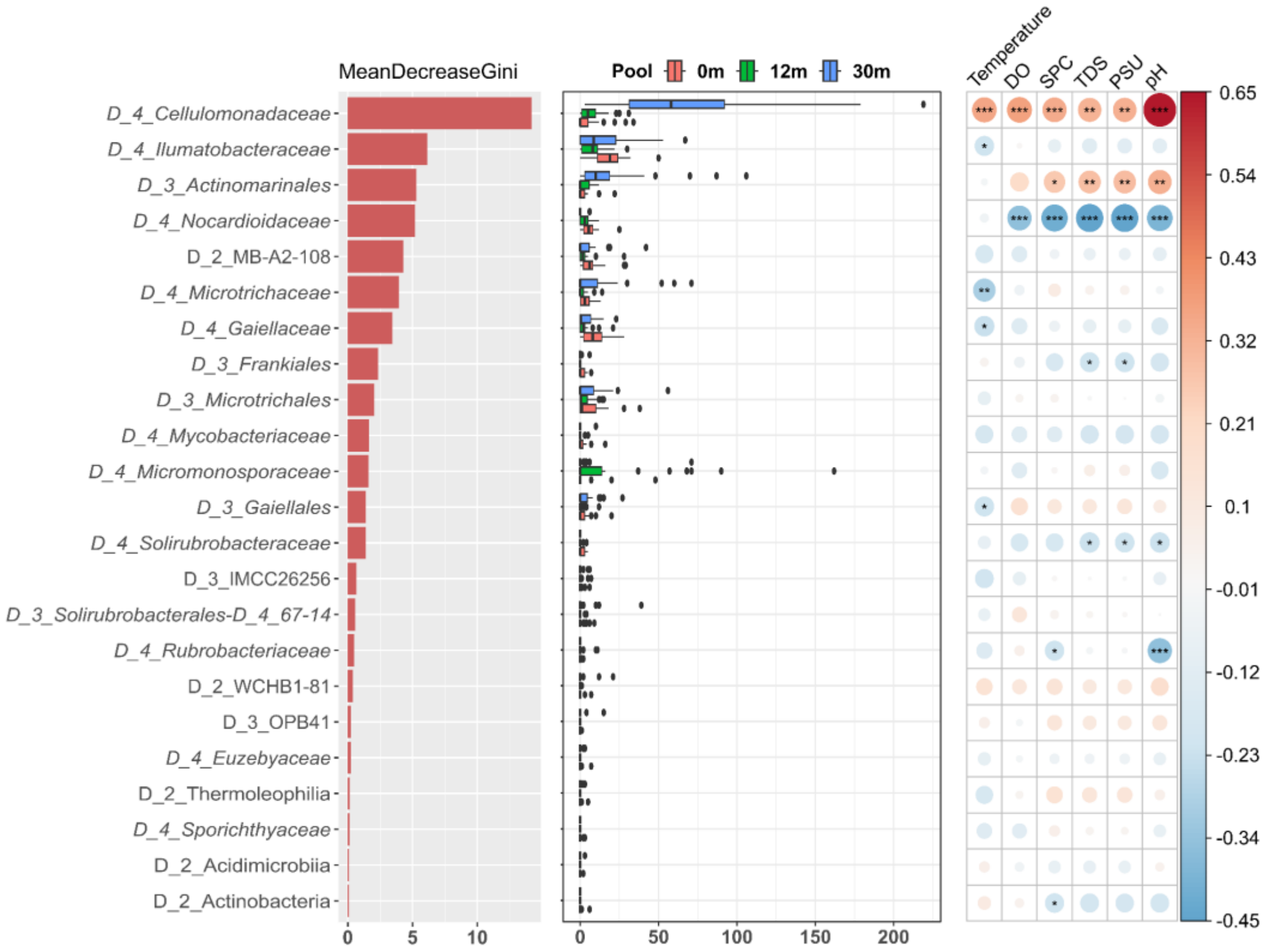


**Fig. S10 Random Forest classification for families of Actinobacteriota and Spearman correlation with environmental factors** The left panel shows the Random Forest analysis results, identifying the most important families within Actinobacteriota across different tide pools. The middle panel presents the read counts of these families, while the right panel illustrates the Spearman rank correlation between read counts and environmental parameters (* p < 0.05; ** p < 0.01; *** p < 0.001).

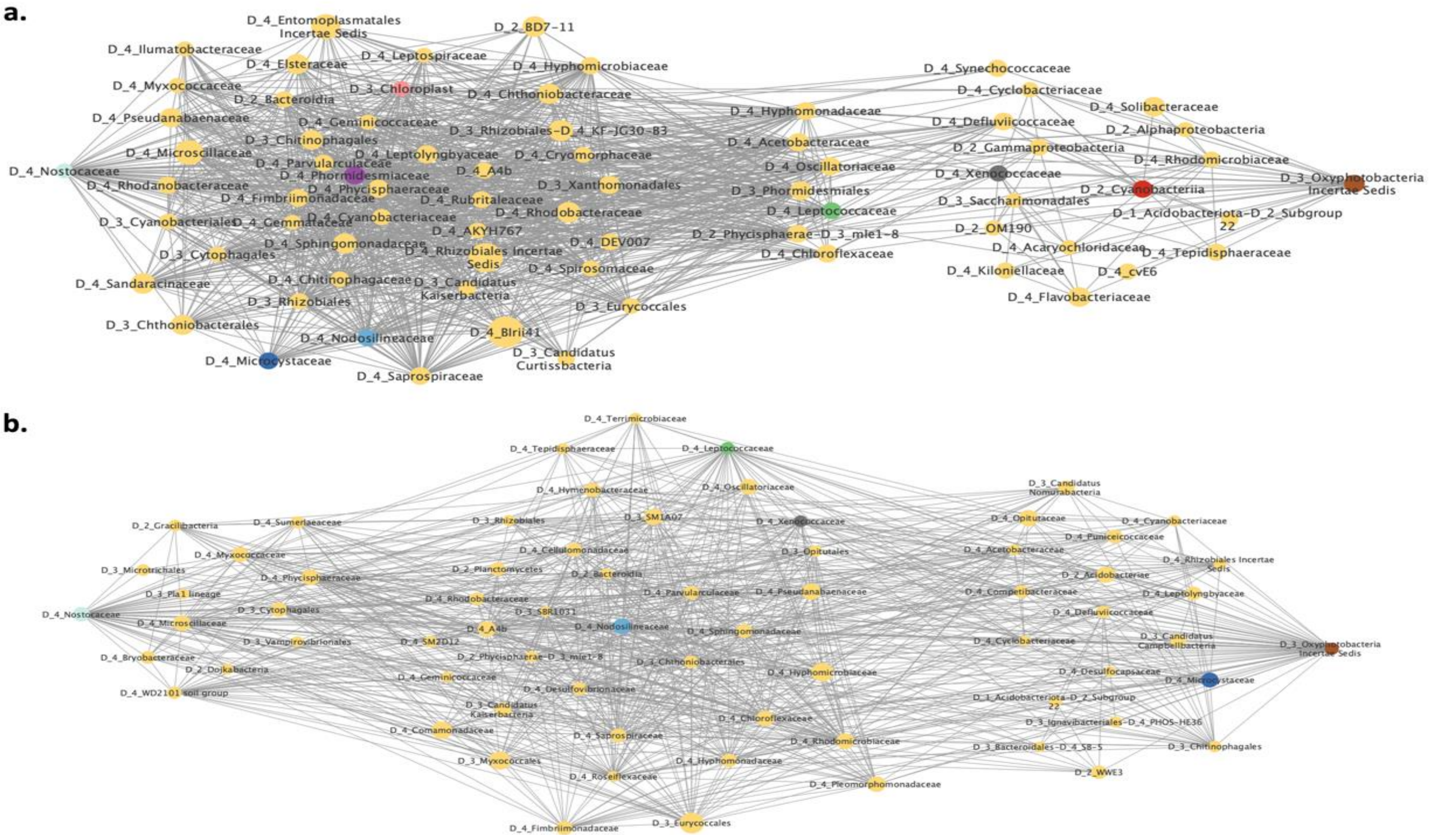
a.
D_4_Entomoplasmatales Incertae Sedis
D_2_BD7-11
D_4_Ilumatobacteraceae
D_4_Leptospiraceae
D_4_Elsteraceae
D_4_Hyphomicrobiaceae
D_4_Myxococcaceae
D_3_Chloroplast
D_4_Chthoniobacteraceae
D_2_Bacteroidia
D_4_Synechococcaceae
D_4_Cyclobacteriaceae
D_4_Pseudanabaenaceae
D_4_Geminicoccaceae
D_4_Hyphomonadaceae
D_4_Solibacteraceae
D_3_Rhizobiales-D_4_KF-JG30-B3
D_4_Defluviicoccaceae
D_3_Chitinophagales
D_2_Alphaproteobacteria
D_4_Acetobacteraceae
D_4_Microscillaceae
D_4_Parvularculaceae
D_4_Leptolyngbyaceae
D_4_Cryomorphaceae
D_2_Gammaproteobacteria
D_4_Rhodomicrobiaceae
D_4_Nostocaceae
D_4_Phormidesmiaceae
D_4_A4b
D_4_Oscillatoriaceae
D_4_Xenococcaceae
D_3_Oxyphotobacteria Incertae Sedis
D_4_Rhodanobacteraceae
D_3_Xanthomonadales
D_4_Phycisphaeraceae
D_3_Phormidesmiales
D_2_Cyanobacteriia
D_4_Fimbriimonadaceae
D_4_Rubritaleaceae
D_3_Saccharimonadales
D_4_Leptococcaceae
D_1_Acidobacteriota-D_2_Subgroup 22
D_4_Cyanobacteriaceae
D_4_Rhodobacteraceae
D_3_Cyanobacteriales
D_4_Gemmataceae
D_2_OM190
D_4_AKYH767
D_2_Phycisphaerae-D_3_mle1-8
D_4_Sphingomonadaceae
D_4_DEV007
D_4_Acaryochloridaceae
D_4_Tepidisphaeraceae
D_3_Cytophagales
D_4_Rhizobiales Incertae Sedis
D_4_Chloroflexaceae
D_4_Spirosomaceae
D_4_Kiloniellaceae
D_4_cvE6
D_4_Sandaracinaceae
D_4_Chitinophagaceae
D_3_Candidatus Kaiserbacteria
D_4_Flavobacteriaceae
D_3_Rhizobiales
D_3_Eurycoccales
D_3_Chthoniobacterales
D_4_Blrii41
D_4_Nodosilineaceae
D_4_Microcystaceae
D_3_Candidatus Curtissbacteria
D_4_Saprospiraceae
b.
D_4_Terrimicrobiaceae
D_4_Tepidisphaeraceae
D_4_Leptococcaceae
D_4_Hymenobacteraceae
D_4_Oscillatoriaceae
D_3_Candidatus Nomurabacteria
D_2_Gracilibacteria
D_4_Sumerlaeaceae
D_3_Rhizobiales
D_3_SM1A07
D_4_Xenococcaceae
D_4_Opitutaceae
D_4_Cyanobacteriaceae
D_4_Puniceicoccaceae
D_4_Myxococcaceae
D_4_Cellulomonadaceae
D_3_Opitutales
D_4_Acetobacteraceae
D_4_Rhizobiales Incertae Sedis
D_3_Microtrichales
D_2_Planctomycetes
D_2_Bacteroidia
D_2_Acidobacteriae
D_4_Phycisphaeraceae
D_3_Pla1 lineage
D_4_Parvularculaceae
D_4_Pseudanabaenaceae
D_4_Competibacteraceae
D_4_Leptolyngbyaceae
D_4_Rhodobacteraceae
D_3_Cytophagales
D_3_SBR1031
D_4_Defluviicoccaceae
D_4_Nostocaceae
D_4_Microscillaceae
D_4_A4b
D_4_Nodosilineaceae
D_4_Sphingomonadaceae
D_3_Vampirovibrionales
D_4_SM2D12
D_4_Cyclobacteriaceae
D_3_Candidatus Campbellbacteria
D_3_Oxyphotobacteria Incertae Sedis
D_4_Bryobacteraceae
D_2_Phycisphaerae-D_3_mle1-8
D_3_Chthoniobacterales
D_2_Dojkabacteria
D_4_Hyphomicrobiaceae
D_4_Desulfocapsaceae
D_4_Geminicoccaceae
D_4_Microcystaceae
D_4_WD2101 soil group
D_4_Desulfovibrionaceae
D_1_Acidobacteriota-D_2_Subgroup 22
D_3_Candidatus Kaiserbacteria
D_4_Chloroflexaceae
D_3_Ignavibacteriales-D_4_PHOS-HE36
D_4_Comamonadaceae
D_4_Saprospiraceae
D_4_Rhodomicrobiaceae
D_3_Bacteroidales-D_4_SB-5
D_3_Chitinophagales
D_3_Myxococcales
D_4_Hyphomonadaceae
D_4_Roseiflexaceae
D_2_WWE3
D_4_Pleomorphomonadaceae
D_4_Fimbriimonadaceae
D_3_Eurycoccales

**Fig. S11 Network of bacteria connected to the dominant Cyanobacteria in the tide pools** (a) shows the network in tide pool 0, and (b) displays the network in the tide pool 30. Each circle indicates a bacterial family. The non-yellow circles indicate cyanobacterial family.

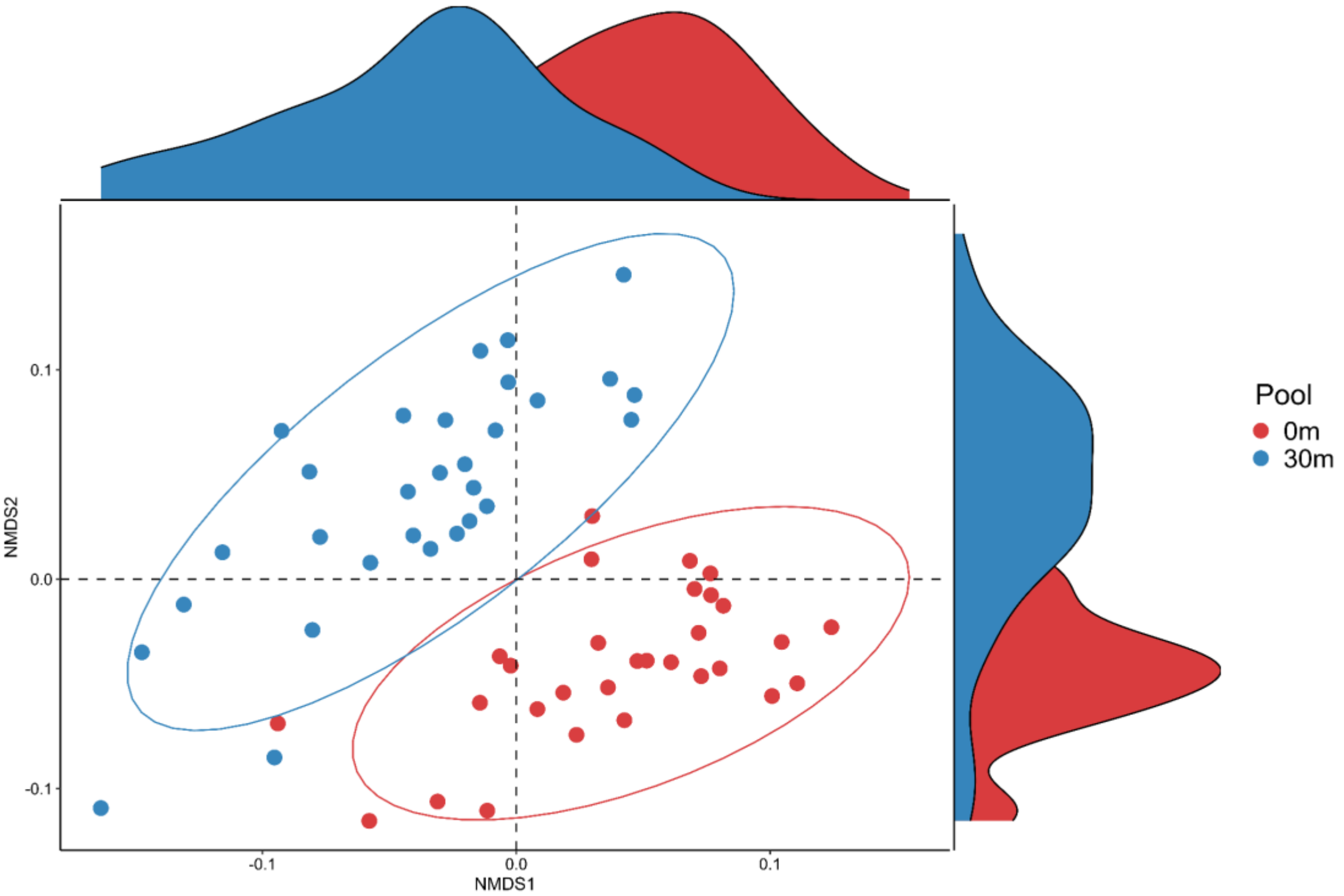


**Fig. S12 NMDS of theoretical metabolic bacterial function** The potential functions of significant bacteria between tide pools 0 and 30 were predicted using PICRUSt2. Different colors represent different tidal pools: red (0m), and blue (30m). (stress: 0.12, PERMANOVA: $p$ = 0.001).

**Table S1 Measured environmental factors across tide pools and sampling months**

| Month | Tide Pool | Depth | Temperature (°C) | DO (%) | SPC (mg/L) | TDS (ppm) | Salinity (psu) | pH |
|---|---|---|---|---|---|---|---|---|
| Jan | 0m | D | 21.2 | 93.2 | 0.76 | 0.494 | 0.37 | 8.11 |
| Jan | 0m | S | 22.8 | 100.7 | 0.68 | 0.446 | 0.34 | 7.87 |
| Jan | 12m | D | 23 | 98.6 | 1.83 | 1.191 | 0.93 | 7.34 |
| Jan | 12m | S | 23.2 | 106.3 | 1.85 | 1.202 | 0.95 | 7.33 |
| Jan | 30m | D | 22.4 | 124 | 4.15 | 2.617 | 2.06 | 8.35 |
| Jan | 30m | S | 24.6 | 111 | 0.95 | 0.6175 | 0.47 | 8.37 |
| Mar | 0m | D | 27.2 | 54.5 | 0.758 | 0.494 | 0.37 | 7.87 |
| Mar | 0m | S | 27.6 | 67.7 | 0.754 | 0.494 | 0.37 | 7.89 |
| Mar | 12m | D | 28.1 | 69.7 | 0.757 | 0.494 | 0.37 | 7.9 |
| Mar | 12m | S | 28.4 | 75.3 | 0.757 | 0.494 | 0.37 | 7.9 |
| Mar | 30m | D | 27.1 | 83.2 | 0.773 | 0.5 | 0.38 | 8.09 |
| Mar | 30m | S | 28.9 | 86.1 | 0.751 | 0.488 | 0.36 | 8.13 |
| Jun | 0m | D | 28.9 | 70 | 0.872 | 0.566 | 0.42 | 7.55 |
| Jun | 0m | S | 29.8 | 80.2 | 0.867 | 0.566 | 0.42 | 7.93 |
| Jun | 12m | D | 29.7 | 73.3 | 0.713 | 0.462 | 0.34 | 8.07 |
| Jun | 12m | S | 30.2 | 72.6 | 0.71 | 0.462 | 0.34 | 8.11 |
| Jun | 30m | D | 30.3 | 74.9 | 0.784 | 0.507 | 0.38 | 8.29 |
| Jun | 30m | S | 30.5 | 76.7 | 0.787 | 0.514 | 0.38 | 8.29 |
| Aug | 0m | D | 29.9 | 103 | 0.677 | 0.442 | 0.33 | 7.85 |
| Aug | 0m | S | 30 | 94.7 | 0.687 | 0.449 | 0.33 | 7.89 |
| Aug | 12m | D | 30.7 | 102.8 | 0.848 | 0.553 | 0.41 | 8.31 |
| Aug | 12m | S | 30.5 | 109.5 | 0.858 | 0.559 | 0.42 | 8.36 |
| Aug | 30m | D | 32.9 | 157.9 | 34.772 | 22.646 | 21.82 | 8.32 |
| Aug | 30m | S | 30.2 | 109.1 | 1.056 | 0.689 | 0.52 | 8.68 |
| Oct | 0m | D | 28.4 | 110 | 0.684 | 0.442 | 0.33 | 7.96 |
| Oct | 0m | S | 28.5 | 105.1 | 0.677 | 0.442 | 0.33 | 7.97 |
| Oct | 12m | D | 29 | 114.2 | 0.671 | 0.436 | 0.32 | 8.07 |
| Oct | 12m | S | 29 | 110.9 | 0.67 | 0.436 | 0.32 | 8.05 |
| Oct | 30m | D | 30.6 | 160 | 44.543 | 29.159 | 28.96 | 8.18 |
| Oct | 30m | S | 29.6 | 120.4 | 2.87 | 1.859 | 1.46 | 8.74 |
